# Cyclic RGD Pentapeptide Cilengitide Enhances Efficacy of Gefitinib on TGF-β1-Induced Epithelial-to-Mesenchymal Transition and Invasion in Human Non-Small Cell Lung Cancer Cells

**DOI:** 10.3389/fphar.2021.639095

**Published:** 2021-03-24

**Authors:** Jisu Jeong, Jiyeon Kim

**Affiliations:** Department of Medical Laboratory Science, School of Health Science, Dankook University, Cheonan, Korea

**Keywords:** Gefitinib, Cilengitide, EMT, TGF-β1, NSCLC, A549

## Abstract

During non-small cell lung cancer (NSCLC) progression, transforming growth factor (TGF)-β mediated epithelial-to-mesenchymal transition (EMT) is an important process leading to high mortality and poor prognosis. The EMT is a fundamental process for morphogenesis characterized by the transformation of cancer cells into invasive forms that can be transferred to other organs during human lung cancer progression. Gefitinib, an epidermal growth factor receptor (EGFR) inhibitor, has shown anti-proliferative effects in EGFR-mutated NSCLC cells and an inhibitory effect on migration and invasion of NSCLC cells to other organs. In this study, we evaluated the combinatorial treatment effect of cilengitide, a cyclic RGD pentapeptide, on TGF-β1-induced EMT phenotype and invasion. Gefitinib suppressed the expression of TGF-β1-induced mesenchymal markers by inhibiting Smad and non-Smad signaling pathways. Cilengitide enhanced the inhibitory effect of gefitinib on TGF-β1-induced expression of mesenchymal markers, phosphorylation of Smad2/3, and invasion of NSCLC A549 cells. We suggested that the use of cilengitide can improve the efficacy of anti-cancer drugs in combination drug-based chemotherapy. These results provide an improved therapeutic strategy for treating and preventing EMT-related disorders, such as NSCLC, lung fibrosis, cancer metastasis, and relapse.

## Introduction

Among the various carcinomas, lung cancer is one of the major causes of cancer-related deaths worldwide and is increasing in incidence each year (Siegel et al., 2011). The 5°years survival rate for lung cancer has not increased significantly, though chemotherapy, radiotherapy, and surgery continue to be studied. In particular, non-small cell lung cancer (NSCLC) accounts for approximately 80–85% of all lung cancers and has high mortality (Siegel et al., 2011). In the progression of NSCLC, the epithelial-to-mesenchymal transition (EMT) is a process whereby closely packed epithelial cells with polarity become more motile and invasive, becoming spindle-shaped mesenchymal cells. Generally, EMT can be seen in the complex process of transformation that epithelial cells undergo to acquire mesenchymal cell characteristics during embryogenesis, development, wound healing, and organ fibrosis (Thiery et al., 2009; Siegel et al., 2011). Among the various factors in cancers, transforming growth factor (TGF)-β is a major cytokine that induces invasion and metastasis through an EMT (Behrens et al., 1989; Buck and Knabbe, 2006; Barcellos-Hoff and Akhurst, 2009; Drabsch and ten Dijke, 2012). Notably, EMT-induced mobility and invasive potential play important roles in cancer metastasis to other organs. The metastatic process in lung cancer is a major cause of mortality and poor inhibition of signaling pathways associated with the EMT process is emerging as a new cancer treatment strategy.

In TGF-β-induced EMT, the binding of TGF-β to transmembrane Ser/Thr receptors TGF-β type I (TβR-I) and type II (TβR-II) results in the phosphorylation of TβR-I and subsequently induces phosphorylation of downstream Smad2 and Smad3 (Heldin et al., 1997; Massague et al., 2005). The Smad2/3 complex, which is phosphorylated in the cytoplasm, recruits Smad4 to the nucleus and binds to transcription factors, such as Snail/Slug and Twist, to activate the EMT-related genes (Massague, 1998; Peinado et al., 2004; Yang et al., 2004). In addition, TGF-β activates the EMT process through the non-Smad signaling pathways, including the Wnt/β-catenin and MEK/ERK signaling pathways in several cancer cells (Galliher et al., 2006; Medici et al., 2008; Chen et al., 2012).

In this study, we examined the anti-cancer effect of gefitinib (trade name “Iressa”), an orally active epidermal growth factor receptor (EGFR) tyrosine kinase inhibitor (TKI) on TGF-β-mediated EMT in NSCLC cells and the effect of its combination with integrin receptor antagonist cilengitide. Gefitinib has been used for the treatment of advanced NSCLC with EGFR-activating mutations in first-line chemotherapy (Kris et al., 2003; Soria et al., 2012). Gefitinib has a high response rate and exhibits disease control in NSCLC patients with active EGFR mutations in the tumors; however, once first-line gefitinib therapy fails, tumor cells become resistant to gefitinib and discovery of the next appropriate regimen becomes very difficult. In addition, the EMT process confers resistance to gefitinib in NSCLC cells (Rho et al., 2009; Suda et al., 2011). To overcome the first-line chemotherapy resistance and enhance drug efficacy, many studies are finding new therapeutic strategies in the treatment of NSCLC patients (Qu et al., 2014; Han et al., 2015; La Monica et al., 2016; Yang and Tam, 2018).

To increase the anti-cancer efficacy of gefitinib and reduce side effects, we evaluated the combinatorial effect of peptide drug cilengitide with gefitinib on cell survival and TGF-β-mediated EMT in NSCLC cells. Cilengitide, a cyclic RGD pentapeptide, is currently in clinical trials for the treatment of several tumors (Mas-Moruno et al., 2010). Because the tripeptide RGD (arginine, glycine, and aspartic acid)-containing molecules can recognize the integrins involved in angiogenesis and tumor metastasis, integrin-targeted RGD-containing peptides or peptidomimetics are candidates for chemotherapy (Plow et al., 2000; Heckmann and Kessler, 2007; Cox et al., 2010). Our results show that combined treatment with cilengitide improves the anti-cancer activity of gefitinib on lung cancer survival and EMT-mediated migration or invasion.

This study provides a new therapeutic strategy, as the combination of integrin-targeted cyclic peptides and TKIs exerts a synergistic effect for the treatment of EMT-related diseases, including human lung cancer, pulmonary fibrosis, and other metastatic cancers.

## Materials and Methods

### Materials

Gefitinib was obtained from Sigma Aldrich (United States), and cilengitide [c(RGDfV)] was synthesized by Dr. Park (CHA Meditech Co., Ltd., Korea) (Supplementary Figure S1). Fetal bovine serum (FBS), Dulbecco’s Modified Eagle’s Medium (DMEM), and antibiotics (100 U/ml penicillin and 100 μg/ml streptomycin) were purchased from Corning Inc. (United States). Recombinant human TGF-β1 was purchased from R&D Systems, Inc. (United States). Antibodies against E-cadherin (#3195), α-smooth muscle actin (α-SMA) (#19245), p-ERK1/2 (#4370), ERK1/2 (#4695), p-EGFR (#3777), EGFR (#4267), and p-Smad2/3 (#8828) were purchased from Cell Signaling Technology, Inc. (United States). Antibodies against Smad2/3 (#sc-133098), vimentin (#sc-6260), N-cadherin (#sc-59987), β-catenin (#sc-7199), horseradish peroxidase (HRP)-conjugated secondary antibodies (#sc-516102 and #sc-2357), and HRP-conjugated actin (#sc-1615) were purchased from Santa Cruz Biotechnology, Inc. (United States).

### Cell Culture and Cell Viability Assay

Human lung fibroblast IMR90 (KCLB No. 10186), bovine pulmonary epithelium CPAE (KCLB No. 10209), NSCLC A549 (KCLB No. 10185), H1650 (KCLB No. 91650), H1299 (KCLB No. 91299), and H358 (KCLB No. 25807) cell lines were obtained from Korean Cell Line Bank. All NSCLC and CPAE cells were maintained in RPMI 1640 medium (Corning, United States) and IMR90 cells in Minimum Essential Medium (MEM; Corning) containing 10% heat-inactivated FBS and 1% antibiotics (100 U/ml penicillin and 100 μg/ml streptomycin). All cells were maintained at 37°C in a humidified atmosphere of 5% CO_2_.

To assess the cell viability, cells (5 × 10^3^ cells/well) were seeded in 96-well plates for 24 h. After incubation, gefitinib or cilengitide were added for 24–72 h in culture media containing 10% FBS. Cell viability was assessed using Cell Counting Kit-8 (Dojindo Molecular Technologies, Japan) according to the manufacturer’s instructions. The absorbance was measured by a Multiscan^TM^ FC microplate photometer (Thermo Fisher Scientific, United States). Cell viability is presented as % of control (untreated cells). Experiments were performed in triplicate.

### Flow Cytometry

A549 cells were seeded at a density of 1.0 × 10^5^ cells/ml in 24-well plates, and incubated for 24 h. Next, the cells were exposed for 24 h to gefitinib or cilengitide in complete media containing 5% FBS. The cells were then harvested and incubated with the MUSE® Annexin V and Dead Cell Assay Kit. To measure the fraction of apoptotic vs. dead cells, we used the MUSE® Cell Analyzer (Merck Millipore, Germany), and data analysis was performed using the MUSE® Annexin V and Dead Cell software module (Merck Millipore, Germany).

### Western Blot Analysis

All cell lysates were prepared using Nuclear and Cytoplasmic Extraction Reagent (Thermo Fisher Scientific, United States). Isolated cytoplasmic or nuclear proteins were separated by SDS-PAGE and transferred to nitrocellulose membranes. Protein expression was detected by specific primary antibodies. Nitrocellulose membranes were incubated with HRP-conjugated secondary antibodies. The band intensities visualized using SuperSignal^TM^ West Femto Maximum Sensitivity Substrate (Thermo Fisher Scientific, United States) and measured using X-ray films and development solution (Fujifilm, Tokyo, Japan). The detected bands were quantified using ImageJ software, and the relative ratios between each sample and the loading control are presented in the figures.

### Immunocytochemistry

A549 cells were incubated on a 1% gelatin-coated slide glass for 24 h. Serum-deprived A549 cells were treated with TGF-β1 and gefitinib for 72 h. Cells were fixed with 4% paraformaldehyde, permeabilized with 0.1% Triton X-100 in PBS, and blocked with 3% BSA in PBS. Expression of vimentin was detected using a primary antibody and visualized with Alexa Fluor 488-conjugated secondary antibodies. Nuclei were counterstained with DAPI. All images were observed by a DeltaVision RT wide-field epifluorescence microscope imaging system and softWoRxs image analysis program (Applied Precision, United States). The relative fluorescence intensity of vimentin was quantified using ImageJ software.

### Invasion Assay

Serum-deprived A549 cells were treated with TGF-β1 (5 ng/ml) and gefitinib or cilengitide or their combination for 48 h. Cells were collected using trypsin-EDTA and resuspended in serum-free medium for counting. Culture medium (30 μL) containing 10% FBS was added to the bottom of a Boyden chamber. After placing over the gelatin-coated membrane filter, the silicone gasket, and the top chamber, the cell suspension (2 × 10^4^ cells/50 μL) was added to the top chamber and incubated at 37°C in 5% CO_2_ for 6 h. The membrane filter was collected, fixed, and stained using the Diff-Quick staining kit (Thermo Fisher Scientific, United States) according to the manufacturer’s instructions. After the filter was dried and stabilized on a glass slide using 30% glycerol solution, the migrated cells were counted in three randomly selected fields at × 400 magnification. All experiments were performed in triplicate. Numbers of invaded cells were presented as an average number of cells per three randomly selected high-power fields (HPFs).

### Quantitative Real-Time PCR Analysis

Total RNA was isolated using the AccuPrep® RNA Extraction Kit (Bioneer Corp., Korea) and the cDNA synthesized from 1 μg of total RNA using oligo (dT) primers (Bioneer Corp., Korea) and the RocketScript^TM^ Reverse Transcriptase Kit (Bioneer Corp., Korea). qRT-PCR was performed using ExcelTaq 2X Q-PCR Master Mix (SMOBiO, Taiwan) and the CFX96^TM^ Real-Time System (Bio-Rad, United States). The cycling conditions were as follows: 95°C for 3 min followed by 40 cycles at 95°C for 15°s, 60°C for 30°s, and 72°C for 30°s. The primer sequences are given in Supplementary Table S1 All reactions were run in triplicate, and data were analyzed using the 2^−ΔΔC^_T_ method (Livak and Schmittgen, 2001). The internal standard was GAPDH. Significance was determined by the Student’s *t*-test with GAPDH-normalized 2^−ΔΔC^_T_ values (Livak and Schmittgen, 2001).

### Analysis of Combined Drug Effects

We analyzed the effects of the drug combination using the CalcuSyn software program (Biosoft, Cambridge, United Kingdom). To determine whether the result of treatment with the two compounds was additive or synergistic, we applied the combination index (CI) derived from the median effect principle of Chou and Talalay (1984). The CI was calculated using the formula published by Zhao et al. (2004). A CI of 1 indicated an additive effect between the two compounds, a CI > 1 indicated antagonism, and a CI < 1 indicated synergism.

### Statistical Analysis

Data are presented as the mean ± SD of at least three independent experiments. Significant differences were evaluated by one-way ANOVA with post-hoc Tukey HSD test. *p* < 0.05 was considered significant.

## Results

### Gefitinib Inhibits NSCLC Cell Proliferation and the TGF-β1-Induced EMT Phenotype

**FIGURE 1:**
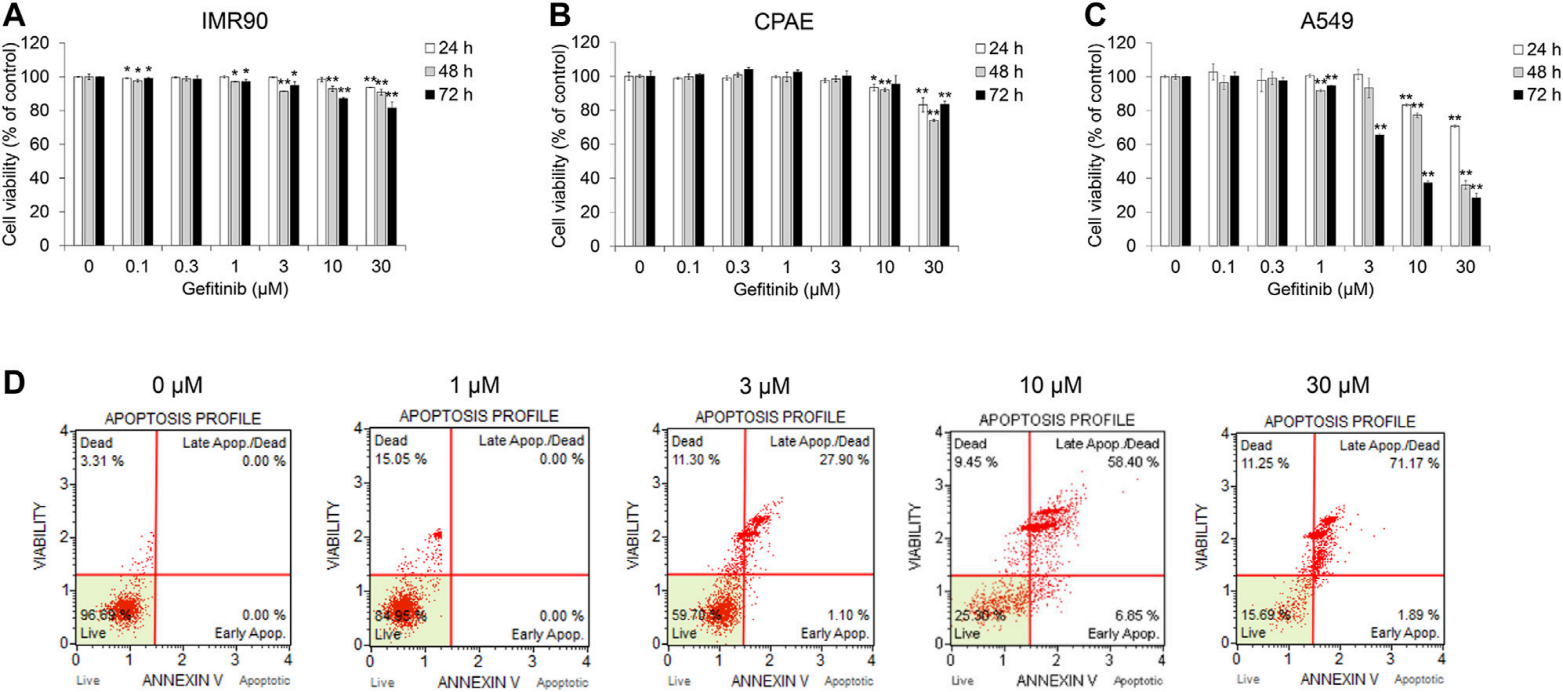
Gefitinib inhibits proliferation of human NSCLC A549 cells. **(A)** A549, **(B)** CPAE and **(C)** IMR90 cells were treated with gefitinib for 24–72 h. After incubation, cell viability was measured by the CCK-8 assay. **(D)** A549 cells were incubated with gefitinib for 72 h, and then the populations of apoptotic and dead cells were analyzed using the MUSE Cell Analyzer. Experiments were performed in triplicate. Data represent mean ± SD. *p < 0.05, **p < 0.01 versus untreated control.

Before evaluating the effect of gefitinib on the TGF-β1-induced EMT, we investigated cell viability in NSCLC A549, normal lung fibroblast IMR90 and normal epithelial cell CPAE cells. Cell growth was inhibited at gefitinib concentrations >1 μM, but the cell viability of IMR90 and CPAE cells was found to have less proportion of dead cells than in A549 cells (Figures 1A–C). In addition, we confirmed that gefitinib induces apoptosis (Figure 1D) and inhibits the proliferation of other NSCLC cells (H1650, H1299, and H358) (Supplementary Figures S4–S6).

**FIGURE 2:**
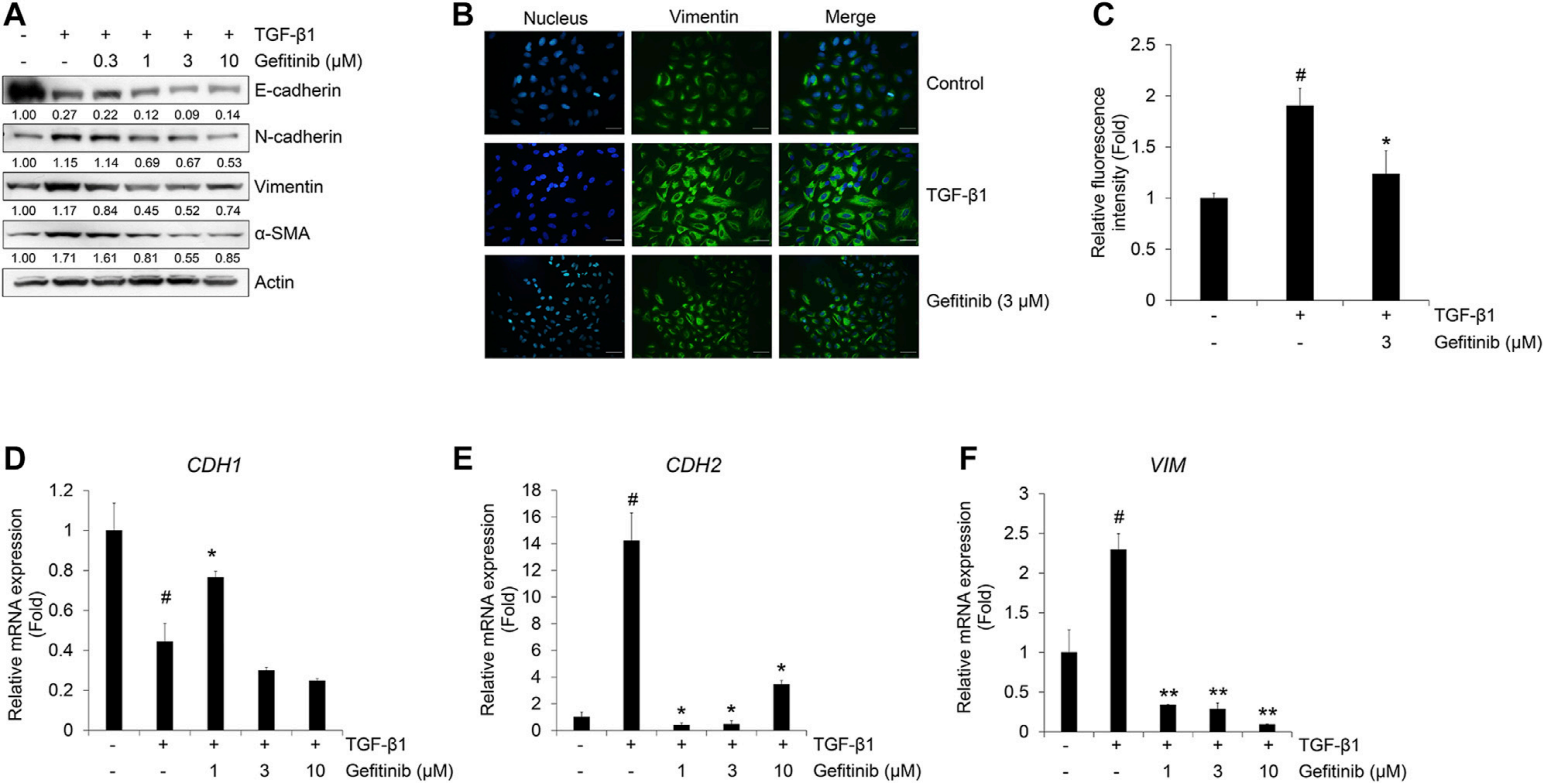
Gefitinib inhibits TGF-β1-induced expression of EMT markers. **(A** and **B)** Serum-deprived A549 cells were treated with TGF-β1 (5 ng/mL) or in combination with gefitinib for 72 h. **(A)** Protein expression of epithelial marker (E-cadherin) and mesenchymal markers (N-cadherin, vimentin, and α-smooth muscle actin) was determined by Western blot analysis. Actin was used as a loading control. **(B** and **C)** TGF-β1-induced expression of vimentin was measured by immunocytochemistry. Nuclei were counterstained with DAPI. Scale bars = 50 µm. **(D–F)** Serum-deprived A549 cells were incubated with TGF-β1 (5 ng/mL) and gefitinib for 24 h. After RNA extraction and cDNA synthesis, we performed qRT-PCR to measure the expression of *CDH1, CDH2*, and *VIM* mRNA using GAPDH as an internal control. ^#^ *p* < 0.01 vs. control; *p < 0.05, **p < 0.01 versus the group treated with TGF-β1 only.

Next, we checked the effect of gefitinib on the TGF-β1-induced EMT phenotype using Western blot analysis and immunocytochemistry. TGF-β1 reduces the expression of epithelial marker E-cadherin, but induces an increase in mesenchymal markers N-cadherin, vimentin, and α-SMA (Kalluri and Weinberg, 2009; Jakobsen et al., 2016). As shown in Figure 2A, gefitinib treatment inhibited TGF-β1-induced increases in N-cadherin, vimentin, and α-SMA. However, the decreased expression of E-cadherin did not fully recover with gefitinib. This explains that gefitinib treatment cannot return cells that have already been transformed into mesenchymal cells by TGF-β1 to their original state, epithelial cell phenotype. This inhibitory effect of gefitinib on TGF-β1-induced expression of the mesenchymal marker was also confirmed by immunocytochemistry. As shown in Figure 2B, TGF-β1-induced morphological changes to mesenchymal cells and enhanced the expression of vimentin. Gefitinib suppressed these morphological changes and vimentin expression. These results indicate that gefitinib exerts an inhibitory effect on TGF-β1-induced mesenchymal phenotype changes and the expression of mesenchymal markers in A549 cells.

We also confirmed these inhibitory effects of gefitinib on the TGF-β1-mediated transcription of epithelial and mesenchymal marker genes *CDH1*, *CDH2*, and *VIM* (encoding E-cadherin, N-cadherin, and vimentin, respectively). TGF-β1 suppressed the expression of *CDH1* mRNA (Figure 2C) and stimulated the expression of *CDH2* (Figure 2D) and *VIM* mRNA (Figure 2E). However, the expression of *CDH1* mRNA slightly recovered at 1 μM, and the expression of *CDH2* and *VIM* mRNA was inhibited at gefitinib concentrations >1 μM. These results indicate that gefitinib concentrations >1 μM exert inhibitory effects on TGF-β1-induced phenotypic changes to mesenchymal cells and related gene expression in A549 cells.

### Gefitinib Inhibits TGF-β1-Induced Smad and Non-Smad Signaling

**FIGURE 3:**
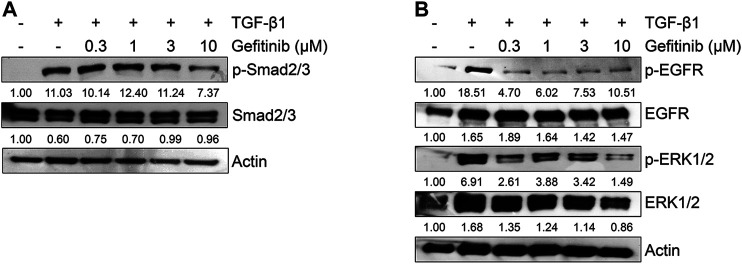
Gefitinib inhibits TGF-β1-induced Smad and non-Smad signaling pathways. Serum-deprived A549 cells were treated with TGF-β1 (5 ng/ml) or in combination with gefitinib for 48 h **(A)** or 72 h **(B)**. Protein expression of the phosphorylated forms of Smad2/3, EGFR, and ERK1/2 and endogenous forms of Smad2/3, EGFR, and ERK1/2 was determined by Western blot analysis. Actin was used as a loading control.

Because the TGF-β1-mediated EMT process is activated by Smad or non-Smad signaling pathways (Heldin et al., 1997; Massague et al., 2005; Galliher et al., 2006; Medici et al., 2008; Chen et al., 2012), we tested the effect of gefitinib on the protein expression of Smad2/3 and MAPK ERK1/2. As shown in Figure 3A, gefitinib suppressed the phosphorylation of Smad2/3. In addition, we measured the phosphorylation of EGFR and downstream MAPK ERK1/2 to determine whether gefitinib inhibits non-Smad signaling. As shown in Figure 3B, TGF-β1-induced phosphorylation of EGFR and ERK1/2 was suppressed by gefitinib. These results suggest that gefitinib has an inhibitory effect on the expression of phosphorylated EGFR and MAPK ERK1/2 that can regulate proliferation and TGF-β-mediated EMT in NSCLC cells.

### Cilengitide Inhibits the TGF-β1-Induced EMT Phenotype in A549 Cells

**FIGURE 4:**
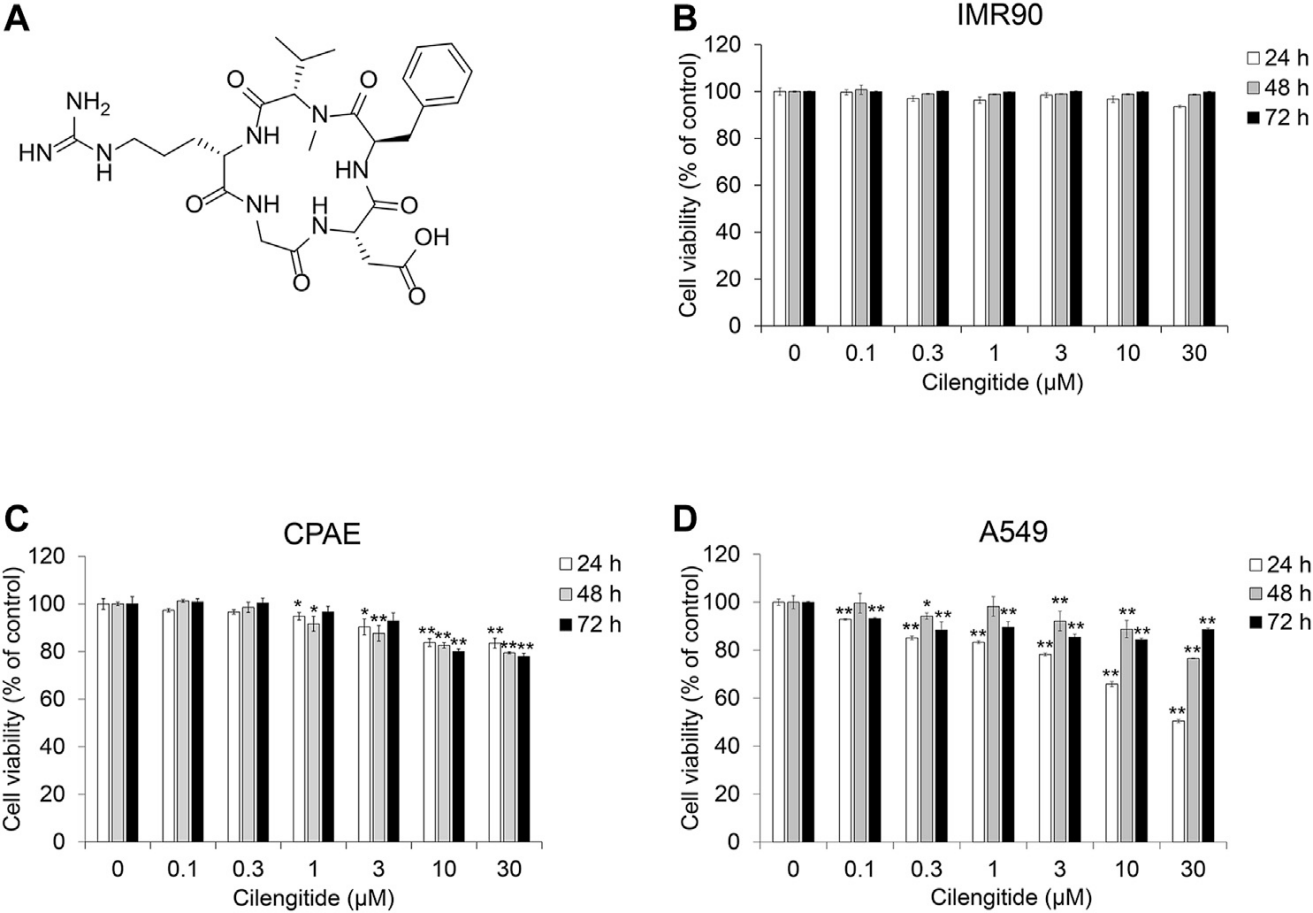
Cilengitide inhibits proliferation of human NSCLC A549 cells. **(A)** Chemical structure of cilengitide. **(B)** IMR90, **(C)** CPAE and **(D)** A549 cells were treated with cilengitide for 24–72 h. After incubation, cell viability was measured by the CCK-8 assay. Experiments were performed in triplicate. Data represent mean ± SD. *p < 0.05, **p < 0.01 vs. untreated control.

To evaluate the effect of cilengitide (Figure 4A and Supplementary Figure S1–S3) on TGF-β1-induced EMT in the presence of gefitinib, we conducted cell viability assays in NSCLC A549 cells, normal fibroblast IMR90 and normal epithelial CPAE cells. As shown in Figures 4B–D, cilengitide suppressed the proliferation of A549 cells, but IMR90 and CPAE cells were not significantly affected. Apoptosis inducing effect and the inhibitory effect of cilengitide on cell growth was confirmed in other NSCLC cells (H1650, H1299, and H358) (Supplementary Figures S4–S7).

**FIGURE 5:**
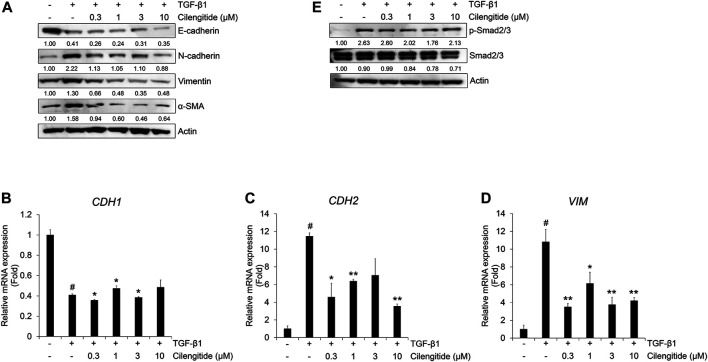
Combination of gefitinib with cilengitide shows enhanced inhibitory effects on TGF-β1-induced EMT expression and Smad signaling. A549 cells were treated with gefitinib (1 µM) and cilengitide (3 µM) individually or in combination, and then incubated with TGF-β1 (5 ng/mL) for 72 h **(A and B)** or 48 h (C) or 24 h **(D and E)**. The expression of EMT markers **(A)** and phosphorylation of Smad2/3 **(C)** were measured by western blot analysis. Actin was used as a loading control. **(B)** The expression of vimentin was measured by immunocytochemistry. Nuclei were counterstained with DAPI. Scale bars = 100 µm. **(D and E)** After RNA extraction and cDNA synthesis, we performed qRT-PCR to measure the expression of *CDH2* and *VIM* mRNA using GAPDH as an internal control. ^#^ *p* < 0.01 vs. control; *p < 0.05, **p < 0.01 vs. the group treated with TGF-β1 only.

We also investigated the effect of cilengitide on the protein expression of TGF-β1-induced EMT markers in A549 cells. Cilengitide did not affect the TGF-β1-induced decrease in E-cadherin, but the expression of N-cadherin, vimentin, and α-SMA was suppressed (Figure 5A). Cilengitide had no effect on the TGF-β1-induced decrease in *CDH1* mRNA expression (Figure 5B), whereas *CDH2* and *VIM* mRNA expression was inhibited by cilengitide treatment independent of dose (Figures 5C,D). In addition, we evaluated the effect of cilengitide on TGF-β1-induced Smad and non-Smad signaling. As shown in Figure 5E, phosphorylation of Smad2/3 by TGF-β1 was slightly suppressed by cilengitide compared to gefitinib treatment. However, TGF-β1-induced phosphorylation of EGFR and ERK1/2 and expression of β-catenin were not inhibited by cilengitide (Supplementary Figure S8). These results indicate that cilengitide exerts weak anti-EMT effects through the inhibition of Smad2/3-mediated signaling in A549 cells.

### Combination of Cilengitide With Gefitinib Exerts a Synergistic Inhibitory Effect on Cell Viability and EMT Phenotype in A549 Cells

Based on the inhibitory effect of gefitinib and cilengitide on cell proliferation and TGF-β1-induced EMT marker expression, we confirmed the combined treatment effect. To verify the effect of cilengitide on cell proliferation and TGF-β1-induced EMT in A549 cells in the presence of gefitinib, we checked the cell viability and mRNA and protein expression of EMT makers. Compared to treatment with gefitinib or cilengitide alone, combined treatment exhibited a synergistic effect on A549 cell proliferation. This result was confirmed by the CI calculated using the raw data (Table 1). Synergistic effects were observed at concentrations >1 μM (24 h), 0.1 μM (48 h), and 0.3 μM (72 h). These synergistic effects were also observed in other NSCLC cells (Supplementary Figures S5–S7; Supplementary Table S2). In other cell lines, combined treatment with ≥0.3 μM resulted in CI values <1.

**TABLE 1:** Combination index (CI) values for the two-drug combination against A549 cell viability.

Incubation time (h)	Gefitinib (μM)	Cilengitide (μM)	CI value
24	0.1	0.1	1.1024
	0.3	0.3	10.0596
	1	1	1.278 × 10^–09^
	3	3	9.27 × 10^–13^
	10	10	1.10 × 10^–19^
	30	30	1.55 × 10^–22^
48	0.1	0.1	0.4465
	0.3	0.3	0.4095
	1	1	0.4208
	3	3	0.5971
	10	10	0.7592
	30	30	0.7081
72	0.1	0.1	1.4553
	0.3	0.3	0.8107
	1	1	0.5699
	3	3	0.6080
	10	10	0.4652
	30	30	0.4658

**FIGURE 6:**
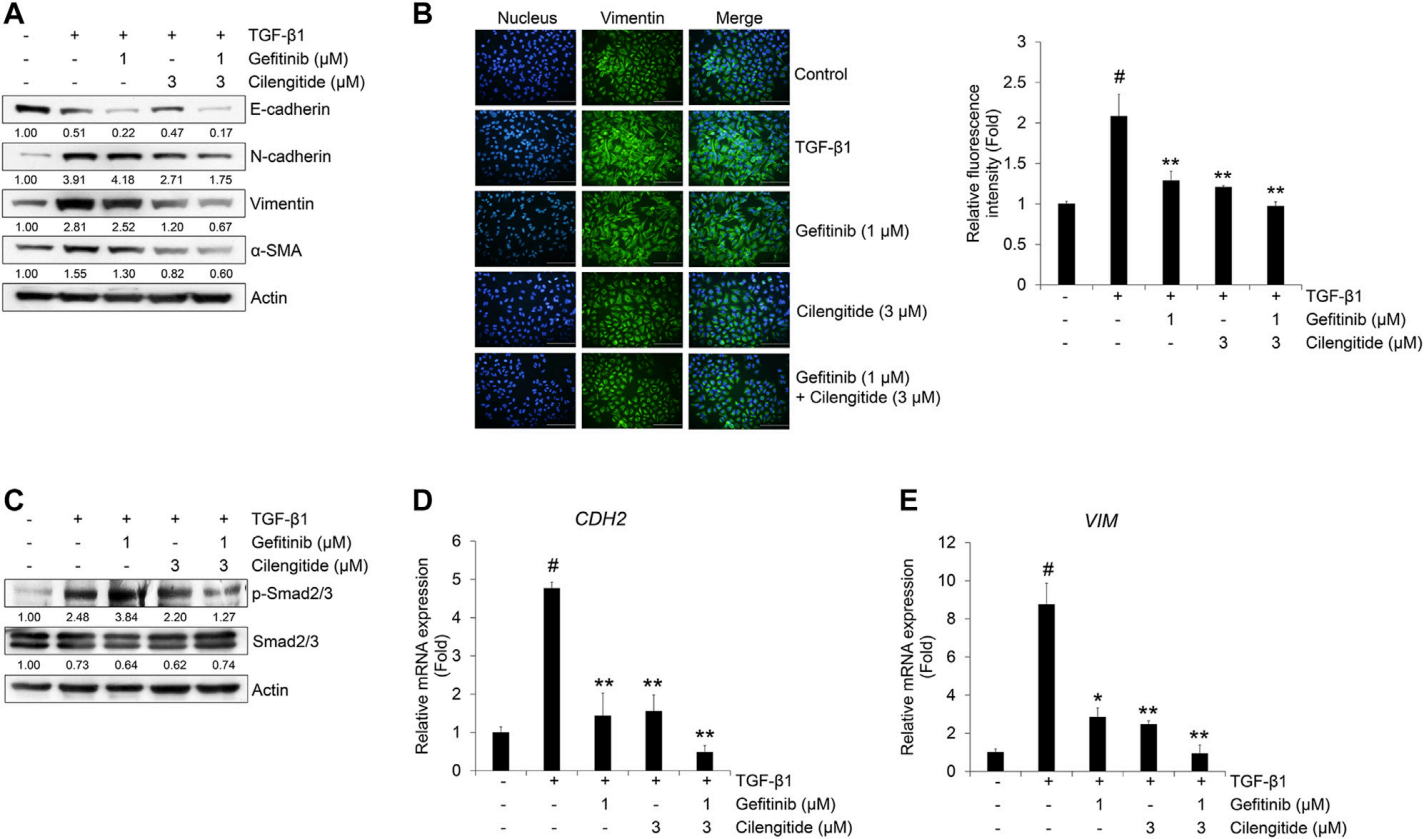
Combination of gefitinib with cilengitide shows enhanced inhibitory effects on TGF-β1-induced EMT expression and Smad signaling. A549 cells were treated with gefitinib (1 μM) and cilengitide (3 μM) individually or in combination, and then incubated with TGF-β1 (5 ng/ml) for 72 h **(A, B)** or 48 h **(C)** or 24 h **(D, E)**. The expression of EMT markers **(A)** and phosphorylation of Smad2/3 **(C)** were measured by western blot analysis. Actin was used as a loading control. **(B)** The expression of vimentin was measured by immunocytochemistry. Nuclei were counterstained with DAPI. Scale bars = 100 μm. **(D, E)** After RNA extraction and cDNA synthesis, we performed qRT-PCR to measure the expression of CDH2 and VIM mRNA using GAPDH as an internal control.^#^ *p* < 0.01 vs. control;*p < 0.05, **p < 0.01 vs. the group treated with TGF-β1 only.

The protein expression of mesenchymal markers N-cadherin, vimentin, and α-SMA was more strongly inhibited by combined treatment with gefitinib and cilengitide than gefitinib alone (Figure 6A). This result was confirmed by immunocytochemistry. As shown in Figure 6B, the expression of vimentin induced by TGF-β1 was strongly suppressed by combined treatment with gefitinib (1 μM) and cilengitide (3 μM). To confirm the synergistic effect of the combined treatment, we checked the Smad and non-Smad signaling. As shown in Figure 6C, TGF-β1-induced phosphorylation of Smad2/3 was more strongly suppressed by combined treatment compared to single treatment with gefitinib or cilengitide. However, non-Smad signaling was not affected by combined treatment (Supplementary Figure S9). In addition, CDH2 and VIM mRNA expression was more strongly suppressed by combined treatment with gefitinib and cilengitide than gefitinib alone (Figure 6D). Treatment with cilengitide alone could not inhibit TGF-β1-induced phosphorylation of Smad2/3, but combined treatment may increase the efficacy of gefitinib to induce NSCLC cell death and inhibit the EMT process through the inhibition of Smad signaling.

### Combination of Cilengitide With Gefitinib Exerts a Synergistic Inhibitory Effect on TGF-β1-Mediated Invasion in A549 Cells

**FIGURE 7:**
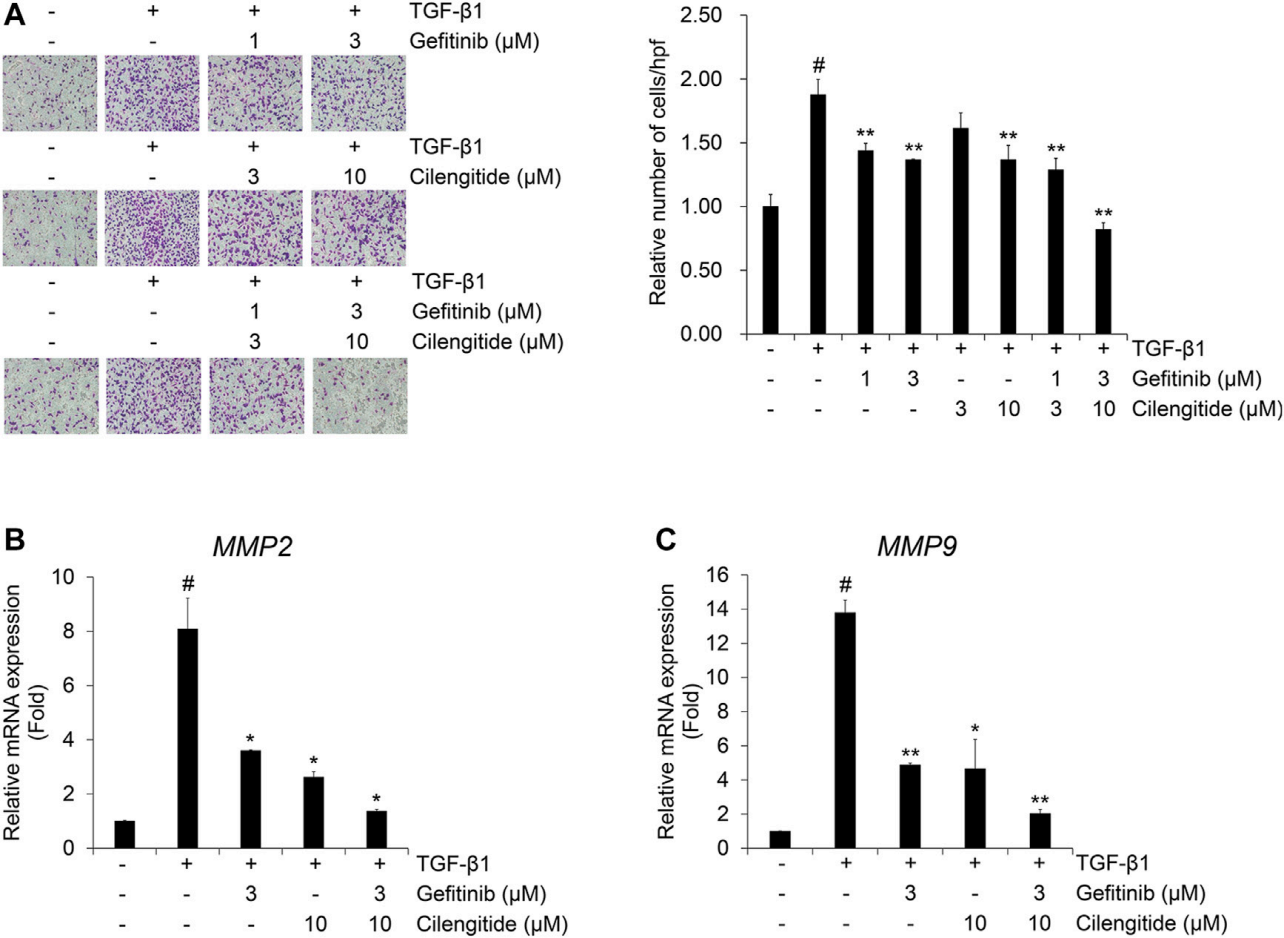
Combination of gefitinib and cilengitide exerts an enhanced inhibitory effect on TGF-β1-induced invasion of A549 cells **(A–C)** Serum-deprived A549 cells were treated with TGF-β1 (5 ng/ml) with or without cilengitide for 48 h. **(A)** The effect of the combination on TGF-β1-induced invasion of A549 cells was evaluated using Boyden chambers. Numbers of invaded cells are presented as an average number of cells per three randomly selected high-power fields (HPFs). **(B, C)** After RNA extraction and cDNA synthesis, we performed qRT-PCR to measure expression of *MMP2* and *MMP9* mRNA using GAPDH as an internal control. Experiments were performed in triplicate. Data represent the mean ± SD of raw results.^#^ *p* < 0.01 vs. control;*p < 0.05, **p < 0.01 vs. treatment with TGF-β1 only.

The combination effect of cilengitide with gefitinib on TGF-β1-induced invasion was also investigated in A549 cells. The TGF-β1-induced invasion of A549 cells across the gelatin-coated membrane was inhibited by gefitinib or cilengitide alone (Figure 7A). This inhibitory effect was enhanced by combined treatment with gefitinib with cilengitide. Quantitative RT-PCR was performed to further investigate the combination treatment effect of gefitinib and cilengitide on the TGF-β1-induced mRNA expression of gelatinases matrix metalloproteinases (MMPs). The extracellular matrix (ECM) disruption contributes to the invasiveness of cancer cells. In this process, MMPs cleave the ECM and contribute to providing a pathway for cancer cells to change their shape to maintain viability and transform them to have invasiveness and migratory characteristics (Najafi et al., 2019). TGF-β1 enhanced MMP2 and MMP9 mRNA expression, and combined treatment with gefitinib and cilengitide more strongly inhibited these transcriptional activities than gefitinib or cilengitide treatment alone (Figures 7B,C). Similar to the effect on the TGF-β1-induced EMT process, these results indicate that combined treatment with gefitinib and cilengitide has strong inhibitory effects on the TGF-β1-induced invasion process and related gene expression.

## Discussion

The EMT process, characterized by loss of epithelial properties and acquisition of mesenchymal features, is a major mechanism for inducing metastatic and invasive changes in cancer cells during lung cancer progression (Kalluri and Weinberg, 2009; Thiery et al., 2009). In addition, the EMT in NSCLC cells stimulates anti-apoptosis signals that confer resistance to chemotherapy, maintaining the survival of cancer cells (Jakobsen et al., 2016). Therefore, suppression of EMT during the progression of NSCLC is an important strategy for preventing cancer cells from having metastatic and invasive characteristics, along with the induction of cancer cell death. In this study, we verified the efficacy of the EGFR-targeted drug gefitinib, which inhibits the TGF-β1-induced EMT in NSCLC cells, and cilengitide, a cyclic pentapeptide that enhances the inhibitory effect of gefitinib.

Several types of RTK inhibitors, such as erlotinib and gefitinib, are used to treat lung cancer, but long-term administration is a major cause of surviving chemotherapy-resistant cells (Jakobsen et al., 2016). In addition, co-administration of small molecule compounds can effectively induce cancer cell death, but may cause side effects, such as cytotoxicity in normal cells. To overcome the limitations of such chemotherapy, recent trends in lung cancer treatment have been to find ways to increase drug efficacy and reduce side effects, including the use of immunotherapy, radiotherapy, and targeted therapy (Yang and Tam, 2018). Among various combination strategies, the use of peptides can reduce side effects that occur with the use of many small molecule drugs. In particular, cyclic peptides have been actively studied as biochemical tools and therapeutics in recent years because of their superior *in vivo* stability, high resistance to exogenous peptidases, and high affinity and selectivity for binding target biomolecules compared to linear peptides (Ong et al., 2017).

To enhance the anti-cancer effect in lung cancer, many studies have demonstrated the possibility of using RGD-containing peptides as anti-cancer drug delivery vectors (Ruoslahti and Pierschbacher, 1987; Ruoslahti, 1996; Xiong et al., 2002; Ruoslahti, 2003; Temming et al., 2005). Because of the high binding affinity and selectivity for integrin α_δ_β_3_ and α _δ_β_5_ in cancer cells, the use of cyclic peptides, such as cRGDfK and cRGDyK, has been one method for delivering therapeutic drugs in cancers (Chen and Chen, 2011; Kulhari et al., 2016). In a previous study, we confirmed that the cyclic pentapeptide containing the RGD motif (cRGDfK) enhances the inhibitory effect of TKI sunitinib on the TGF-β1-induced EMT (Park and Kim, 2020). The cRGDfK not only promotes the NSCLC cell death-inducing effect of gefitinib, but also increases the inhibitory effect on mesenchymal marker mRNA and protein expression.

Based on previous studies, we investigated whether cilengitide (cRGDfV), a derivative of cRGDfK, enhances the inhibitory effect of gefitinib on cell proliferation and the TGF-β1-induced EMT in NSCLC cells. In this study, gefitinib, and cilengitide each exerted inhibitory effects on NSCLC cell proliferation and the TGF-β1-induced EMT process. However, single compound treatment exhibited an inhibitory effect at high concentrations and was found to effectively suppress the EMT at low concentrations when treated in combination. This finding demonstrates that cilengitide, as an integrin-targeting peptide, can enhance the efficacy of gefitinib in NSCLC cells.

Recent studies have suggested new methods to improve the therapeutic efficacy of cancer treatment using combination strategies with cilengitide (Lautenschlaeger et al., 2013; Heiduschka et al., 2014; Vansteenkiste et al., 2015; Wichmann et al., 2017; Zeng et al., 2020). To enhance the anti-cancer effect, clinical trials are investigating the combination of cilengitide with existing cancer drugs (Vansteenkiste et al., 2015; Haddad et al., 2017; Massabeau et al., 2018). Although these clinical studies have demonstrated the efficacy of cilengitide, no studies have been conducted on the mechanism of EMT suppression in NSCLC and its combination with cancer treatment drugs. Thus, the combination of cilengitide with gefitinib may be a new applicable method to modulate growth and invasive changes in EMT-mediated EGFR-resistant NSCLC. However, the consideration for future research is the efficacy of cilengitide in NSCLC depending on the EGFR mutation or wild-type. Drugs that are effective against cells with EGFR mutation, such as gefitinib, and drugs that are not effective should be compared to cilengitide in the EMT process in NSCLC.

New drug development in the modern biopharmaceutical industry is becoming increasingly difficult with increasing R&D time and costs. To reduce these requirements, pharmaceutical companies are pursuing strategies to diversify new drug development methods. Among various methods, drug repositioning is a method of investigating efficacy by applying existing drugs to new diseases (Chong and Sullivan, 2007; Hanusova et al., 2015). The biggest advantage of drug repositioning is that the drugs to be used for treatment have already been evaluated for safety and toxicity. Therefore, this approach can achieve synergistic effects of therapeutic efficacy without the development of new drugs through the co-administration of existing therapeutic agents and cilengitide in NSCLC.

Among the RGD-based cyclic peptides, cilengitide has been actively investigated for anti-cancer efficacy, but studies on other peptides have yet to reach clinical trials. In addition, there have been few results confirming the co-administration effect of small molecule compounds and peptides in lung diseases affected by EMT, including NSCLC. The results of this study suggest that cyclic peptides may be helpful in preventing NSCLC progression and EMT-related lung diseases in the future. We plan to confirm the synergistic effect of cilengitide in the EMT in other lung diseases.

## Data Availability

The datasets presented in this study can be found in online repositories. The names of the repository/repositories and accession number(s) can be found in the article/Supplementary Material.
