# Supplementary material for: Cyclic RGD Pentapeptide Cilengitide Enhances Efficacy of Gefitinib on TGF-β1-Induced Epithelial-to-Mesenchymal Transition and Invasion in Human Non-Small Cell Lung Cancer Cells
Source: Front Pharmacol. 2021 Mar 24;12:639095. doi: 10.3389/fphar.2021.639095 (PMC8104086; doi:10.3389/fphar.2021.639095)

Fig. 2A

A549_Gefitinib_72 h

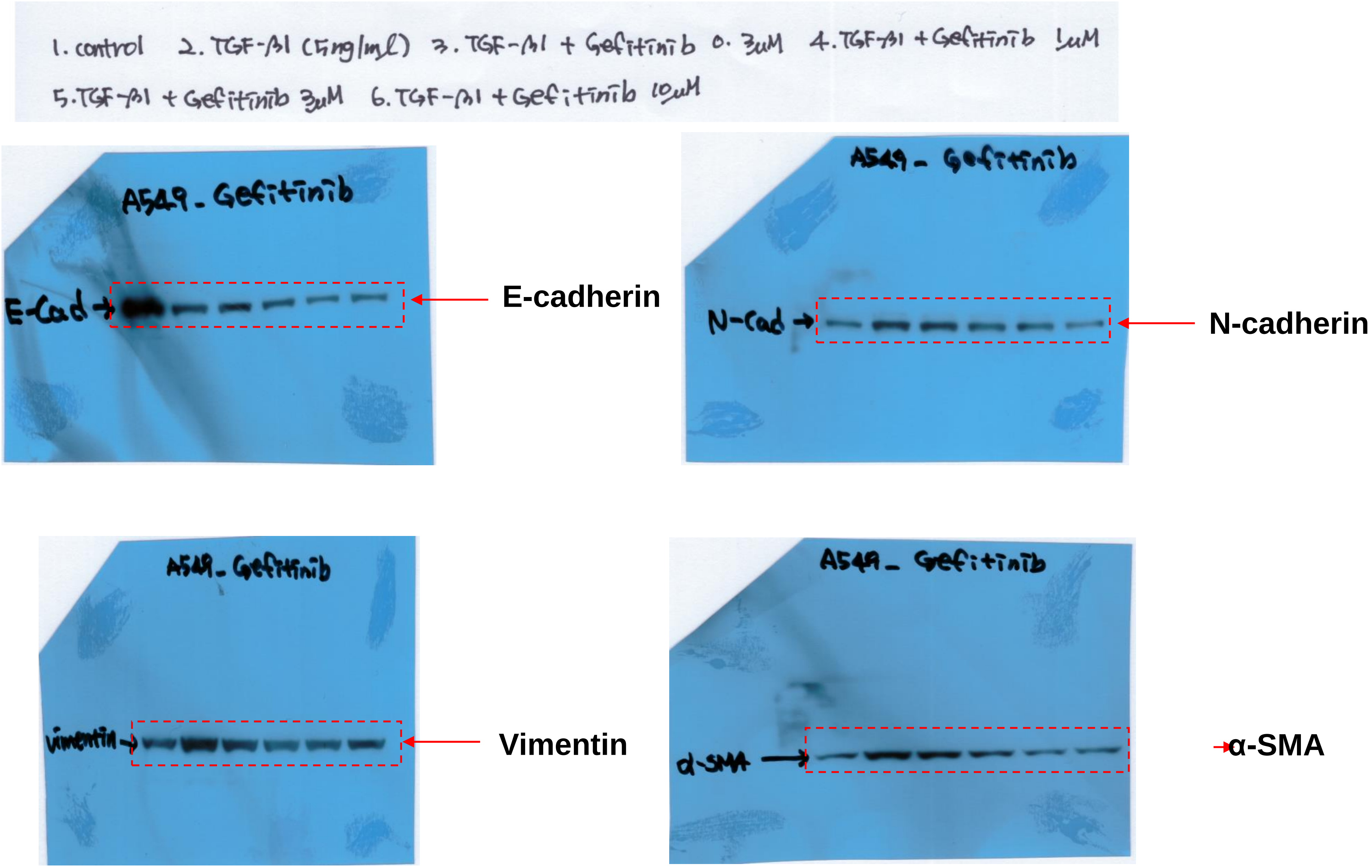

A549_Gefitinib_72 h

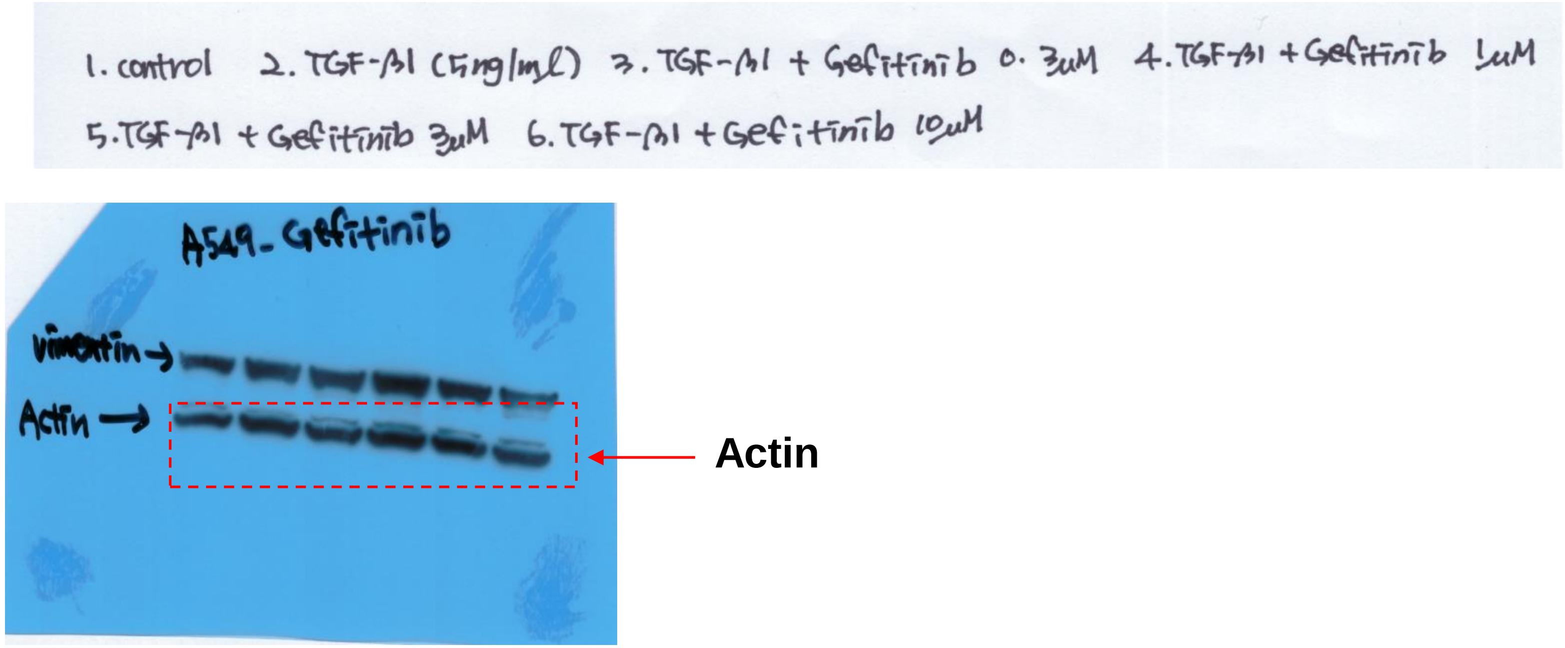

Fig. 3A

A549_Gefitinib_48 h

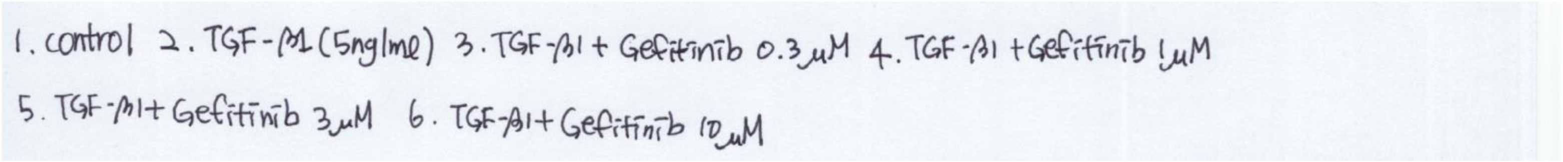

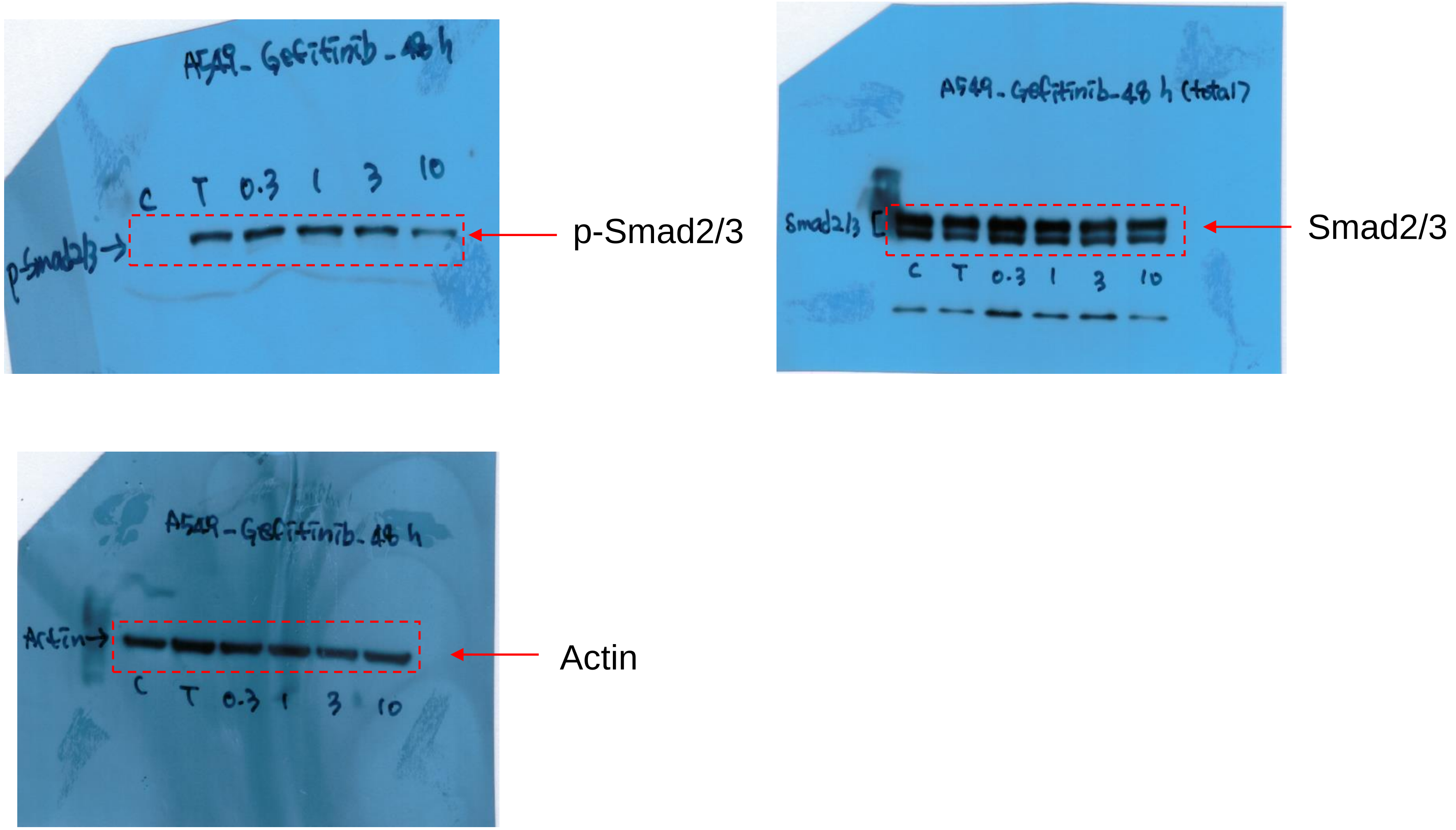

Fig. 3B

A549_Gefitinib_72 h

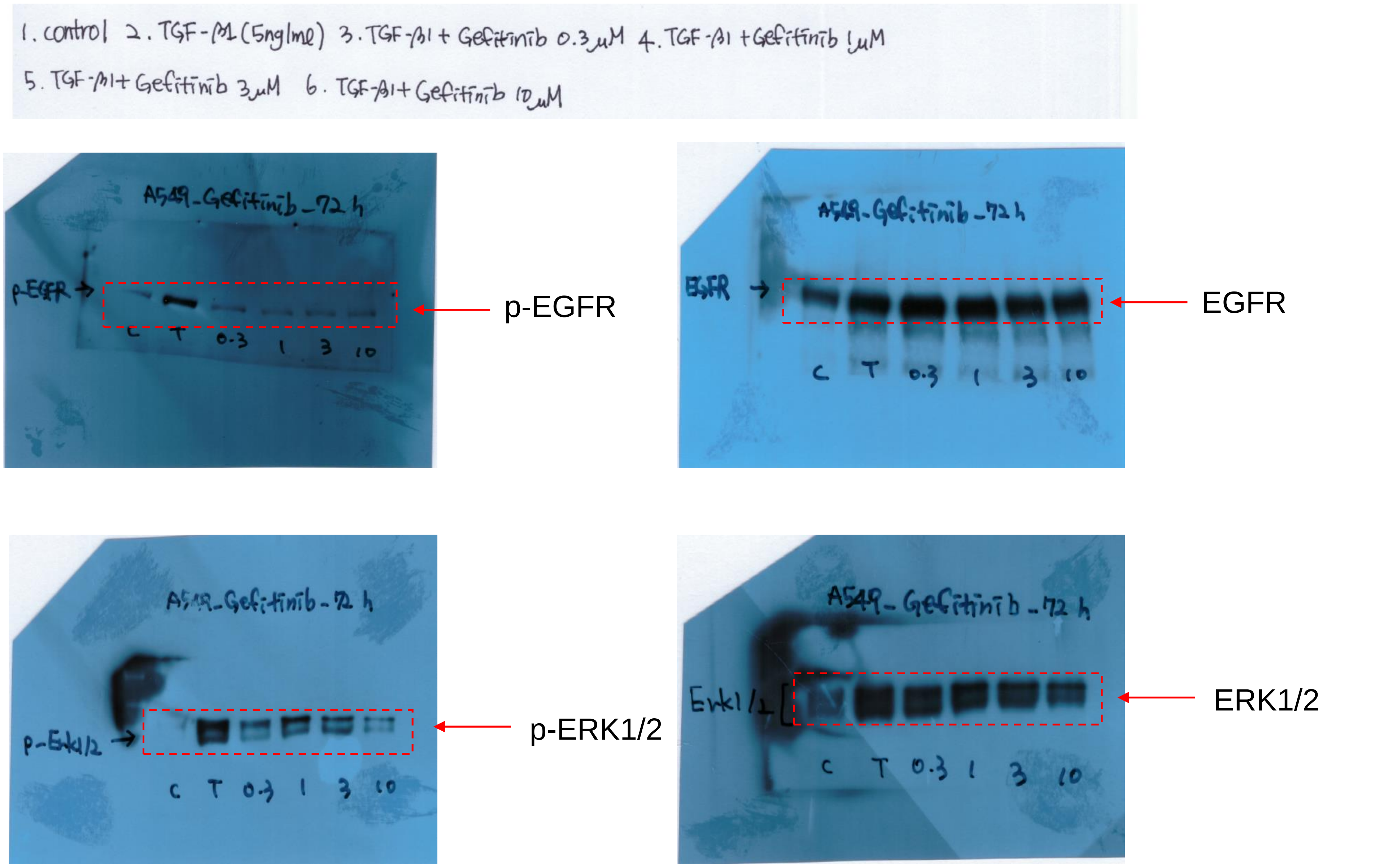

Fig. 3B

A549_Gefitinib_48 h

1. control 2. TGF-β1 (5ng/ml) 3. TGF-β1 + Gefitinib 0.3 μM 4. TGF-β1 + Gefitinib 1 μM
5. TGF-β1 + Gefitinib 3 μM 6. TGF-β1 + Gefitinib 10 μM

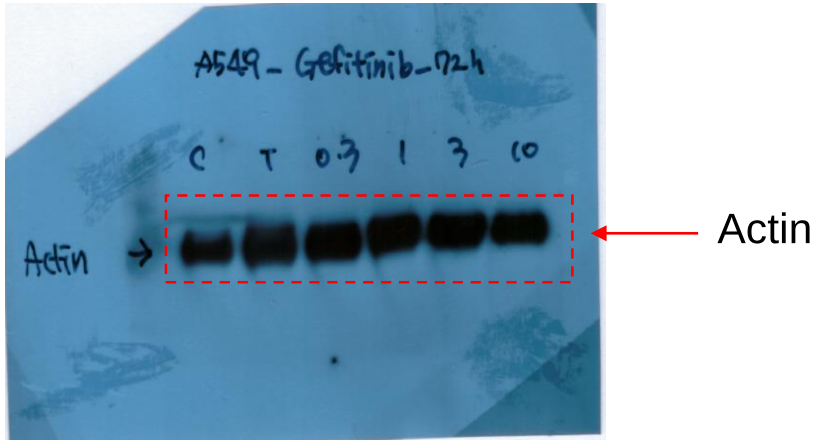

Fig. 5A

A549_Cilengitide_72 h

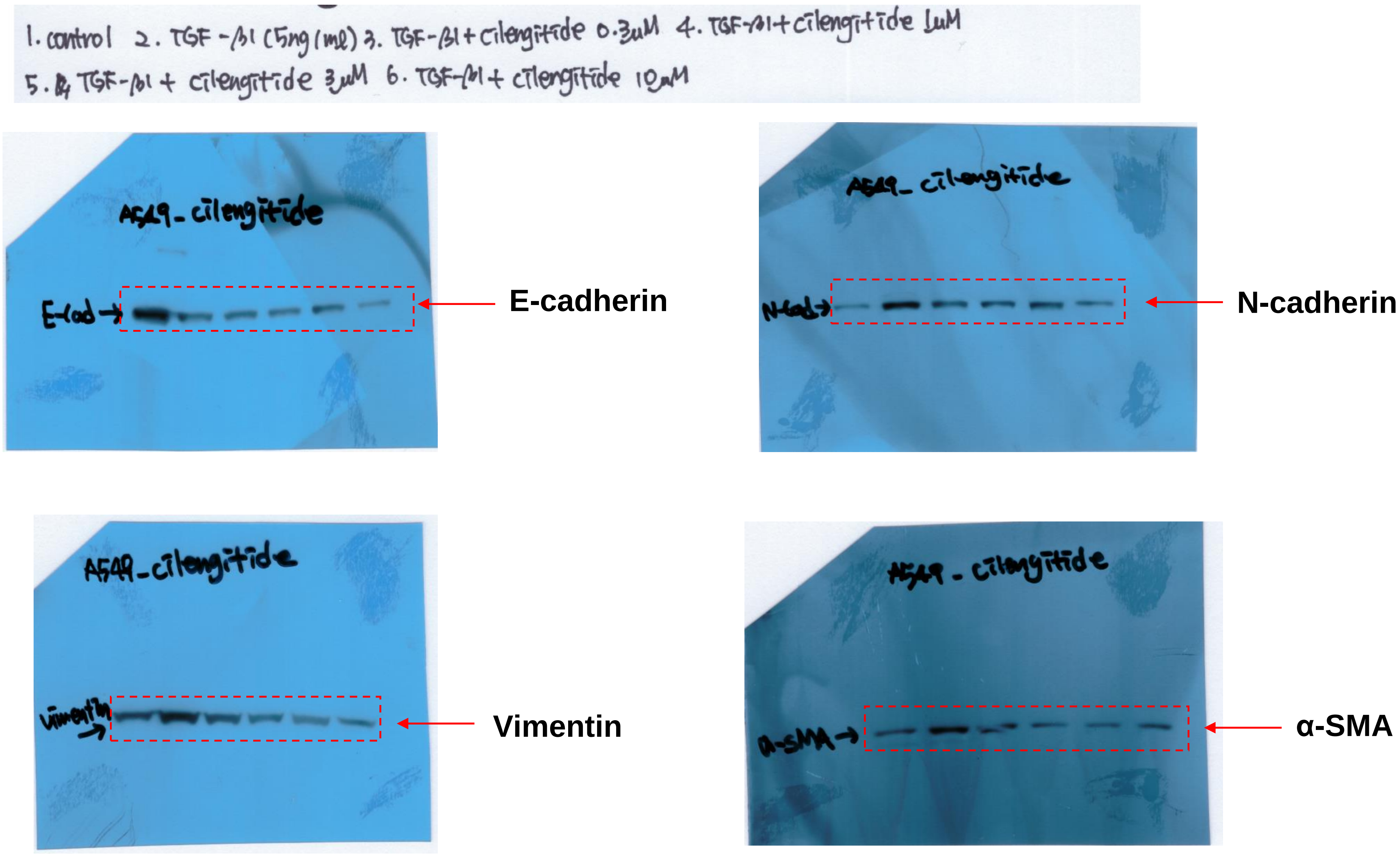

Fig. 5A

A549_Cilengitide_72 h

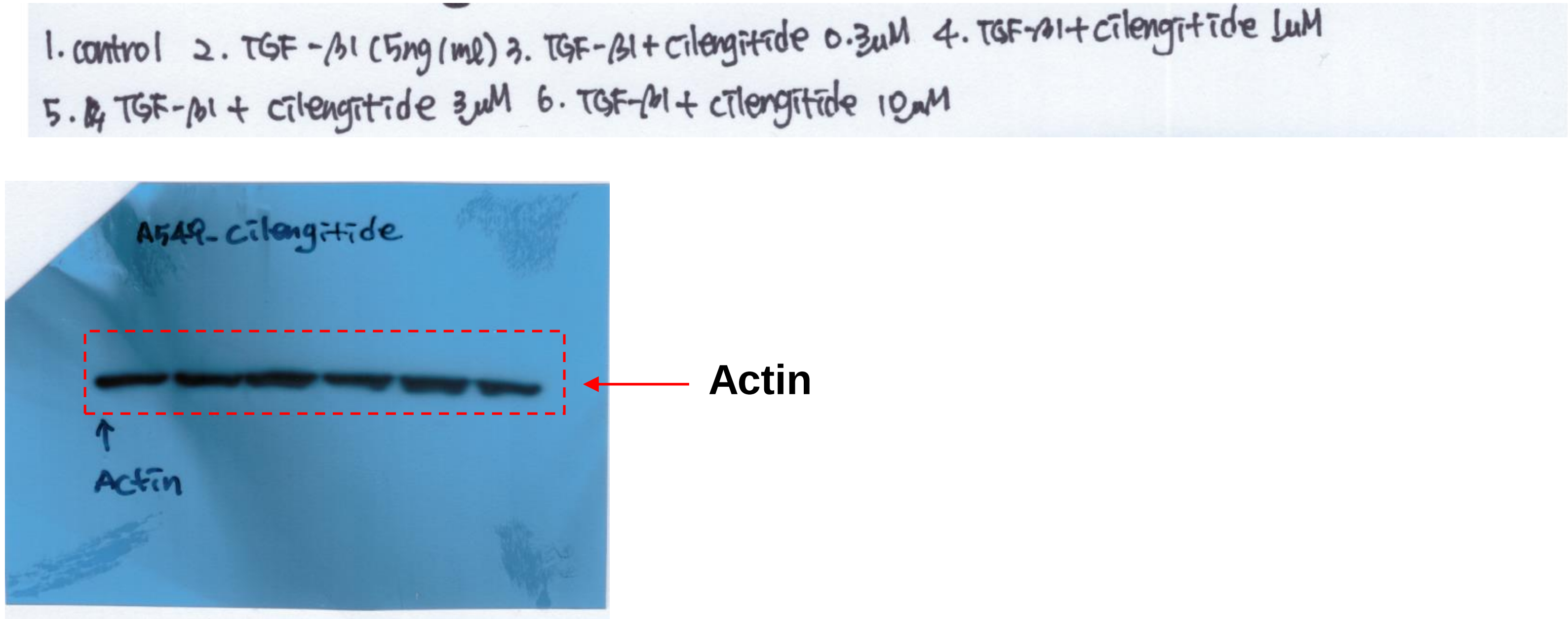

Actin

Fig. 5B

A549_Cilengitide_48 h

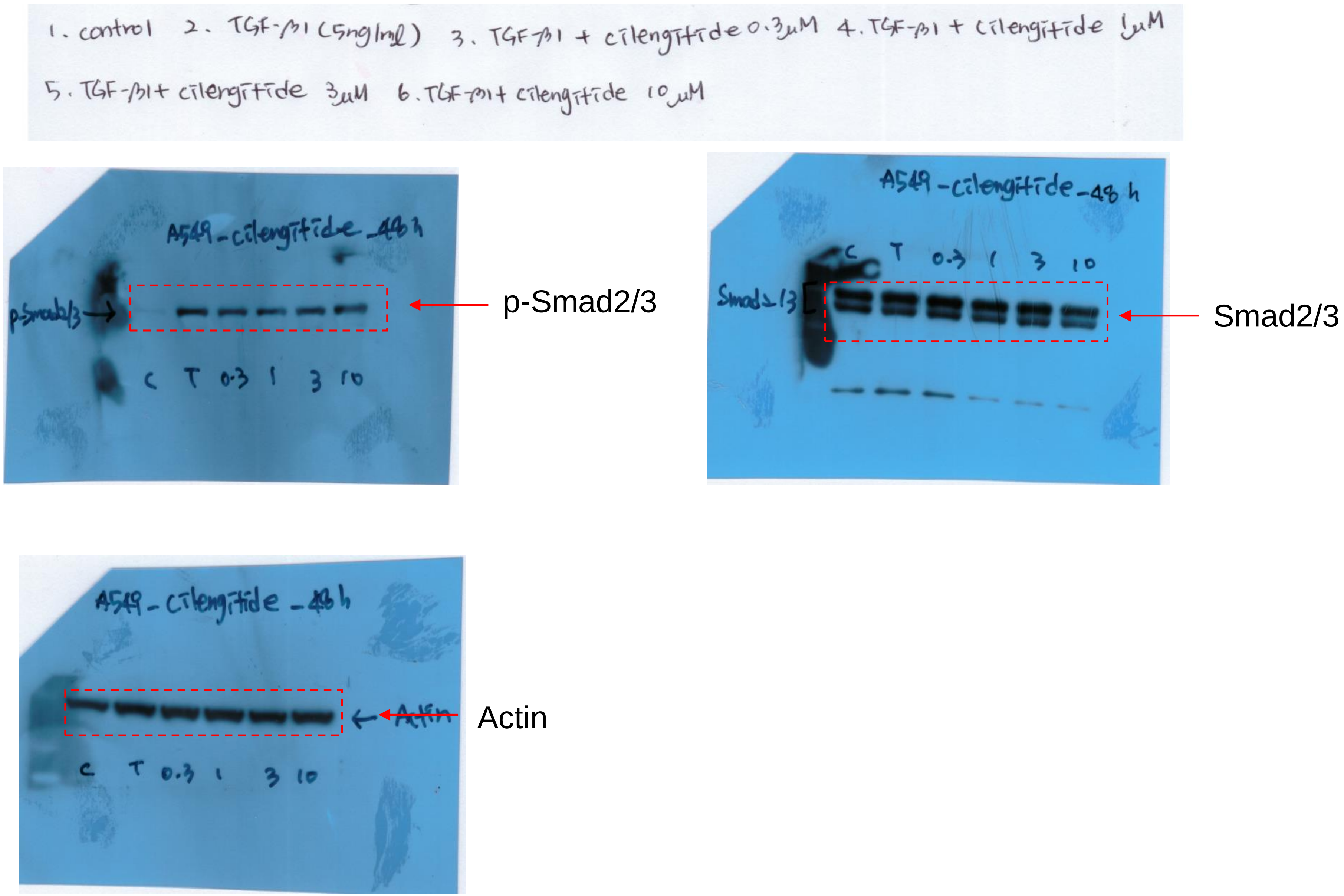

Fig. 6A

A549_Combi (Gefitinib + Cilengitide)

1. control 2. TGF-β1 (5ng/ml) 3. TGF-β1 + Gefitinib 1uM 4. TGF-β1 + cilengitide 3uM
5. TGF-β1 + Gefitinib 1uM + Cilengitide 3uM

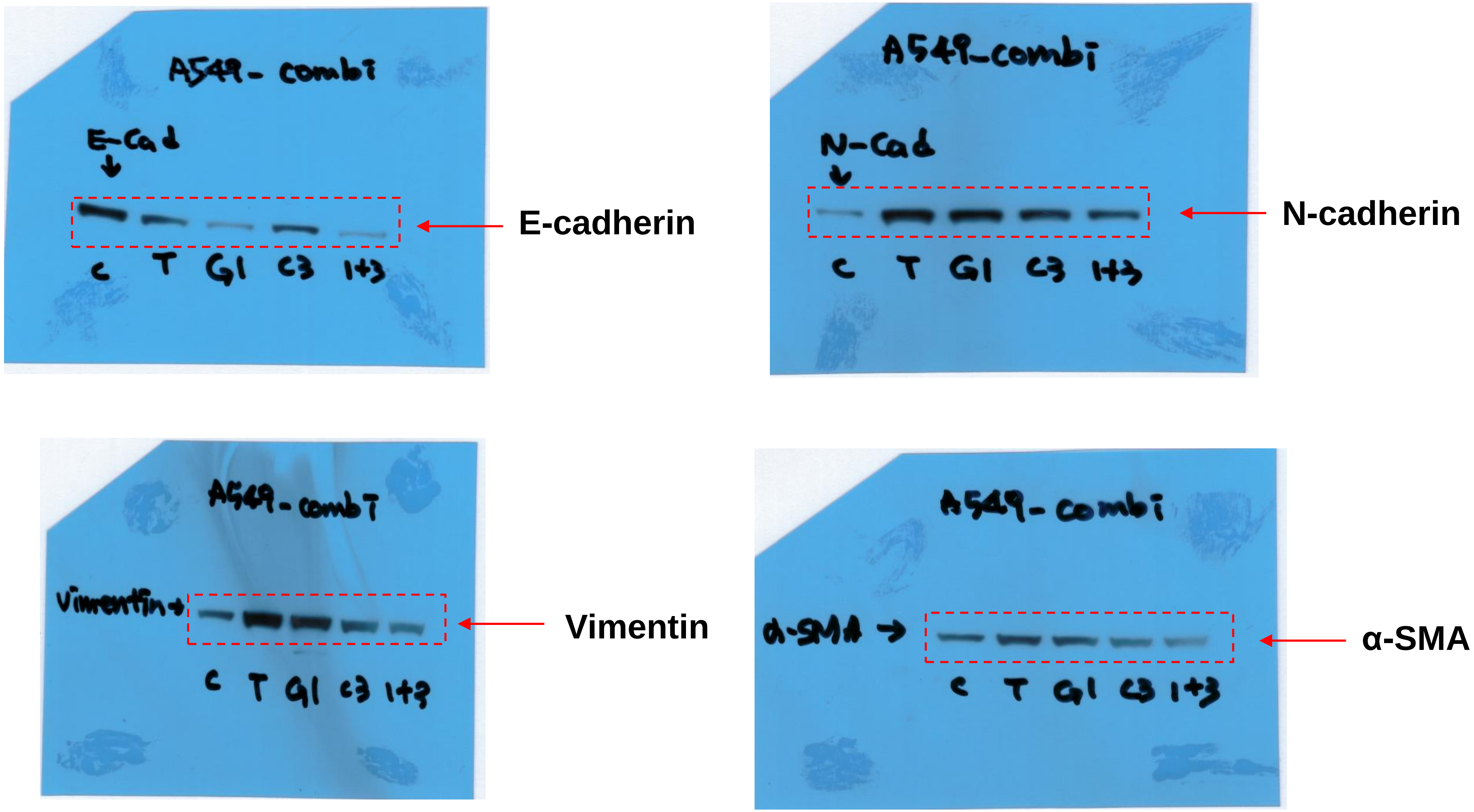

Fig. 6A

A549_Combi (Gefitinib + Cilengitide)

1. control 2. TGF-β1 (5ng/ml) 3. TGF-β1 + Gefitinib 1uM 4. TGF-β1 + Cilengitide 3uM
5. TGF-β1 + Gefitinib 1uM + Cilengitide 3uM

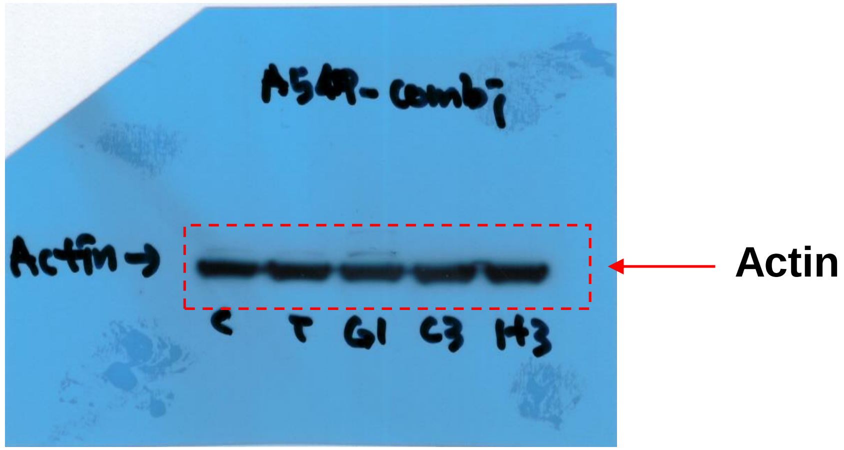

Fig. 6C

A549_Combi (Gefitinib + Cilengitide)

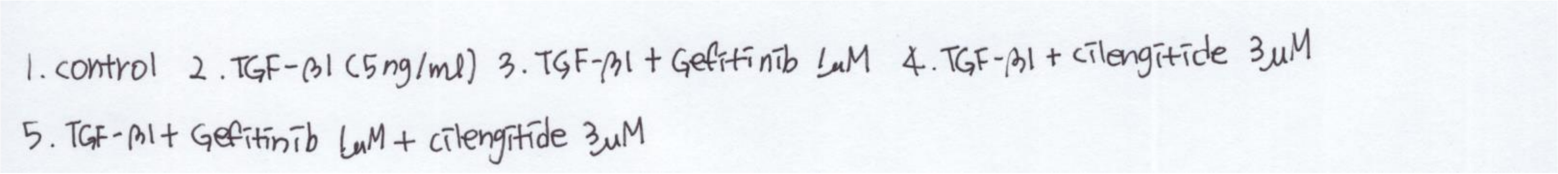

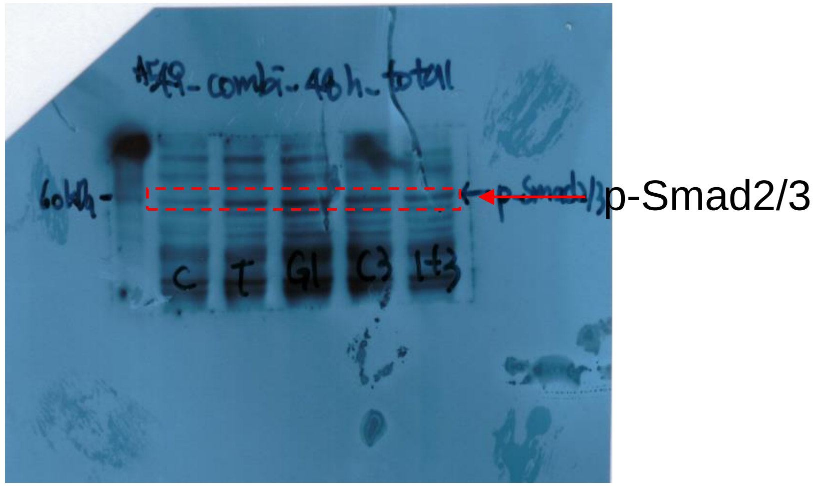

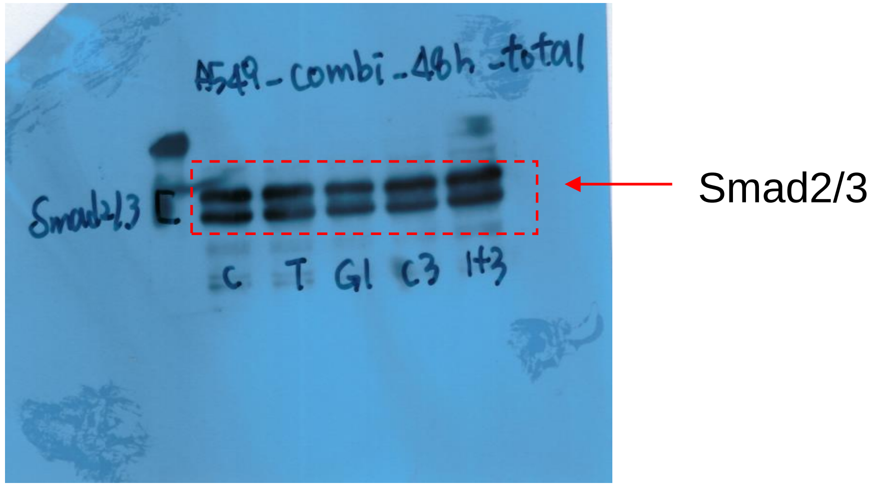

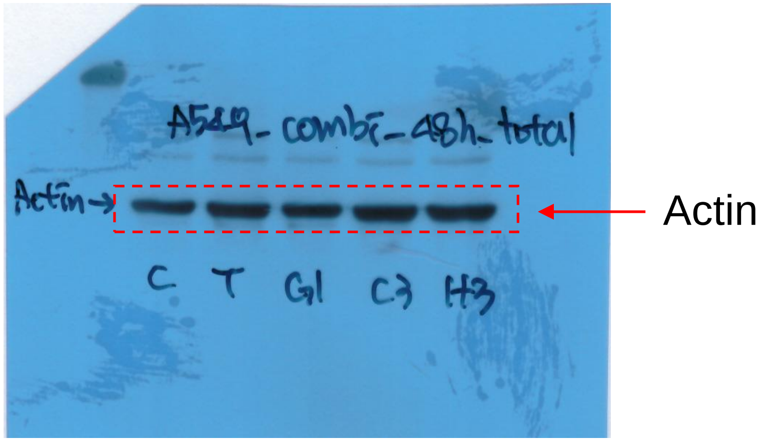

Fig. S7A

A549_Cilengitide_72 h

1. control 2. TGF-β1 (5ng/ml) 3. TGF-β1 + cilengitide 0.3μM 4. TGF-β1 + cilengitide 1μM
5. TGF-β1+ cilengitide 3μM 6. TGF-β1+ cilengitide 10μM

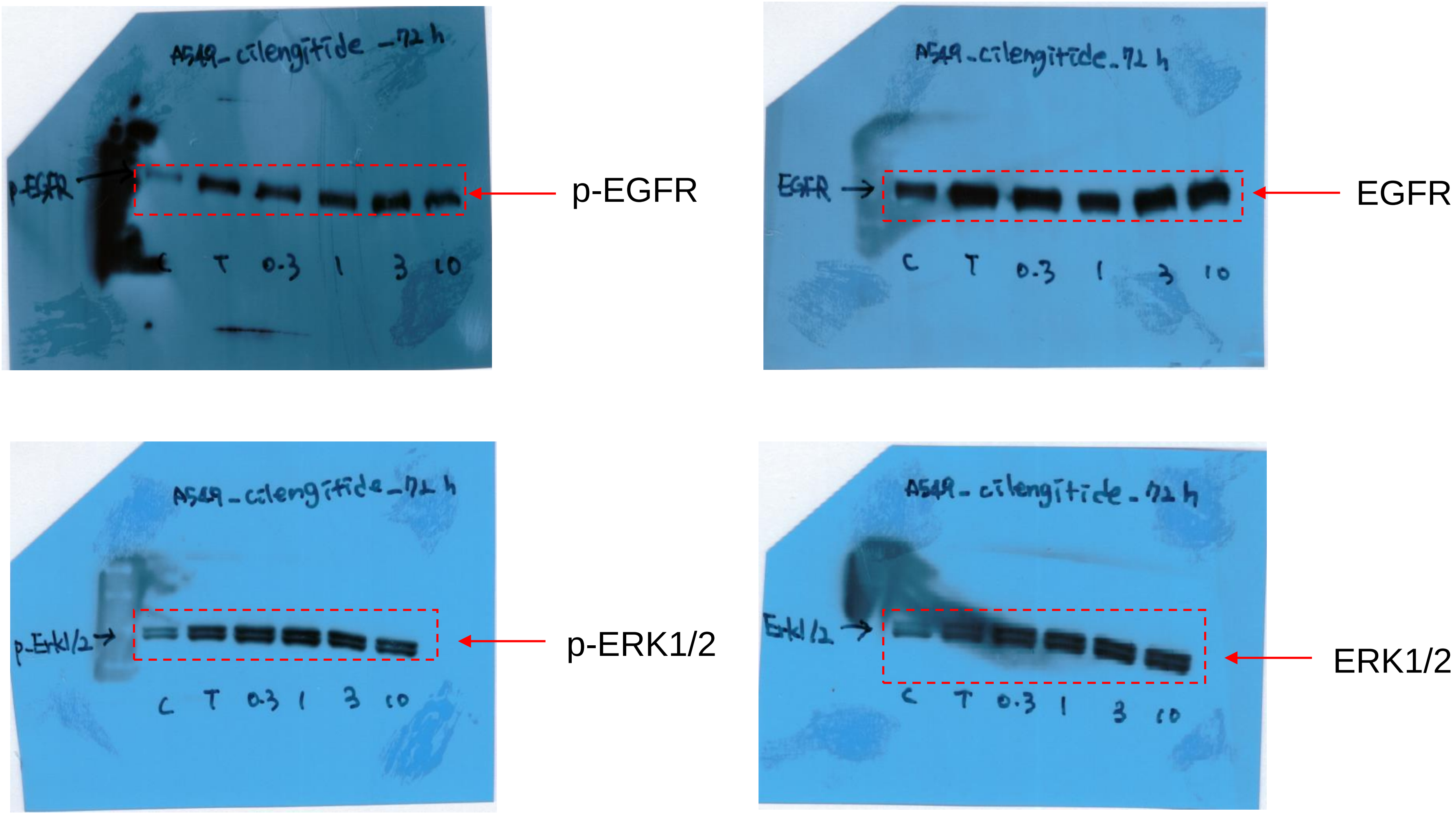

Fig. S7A

A549_Cilengitide_72 h

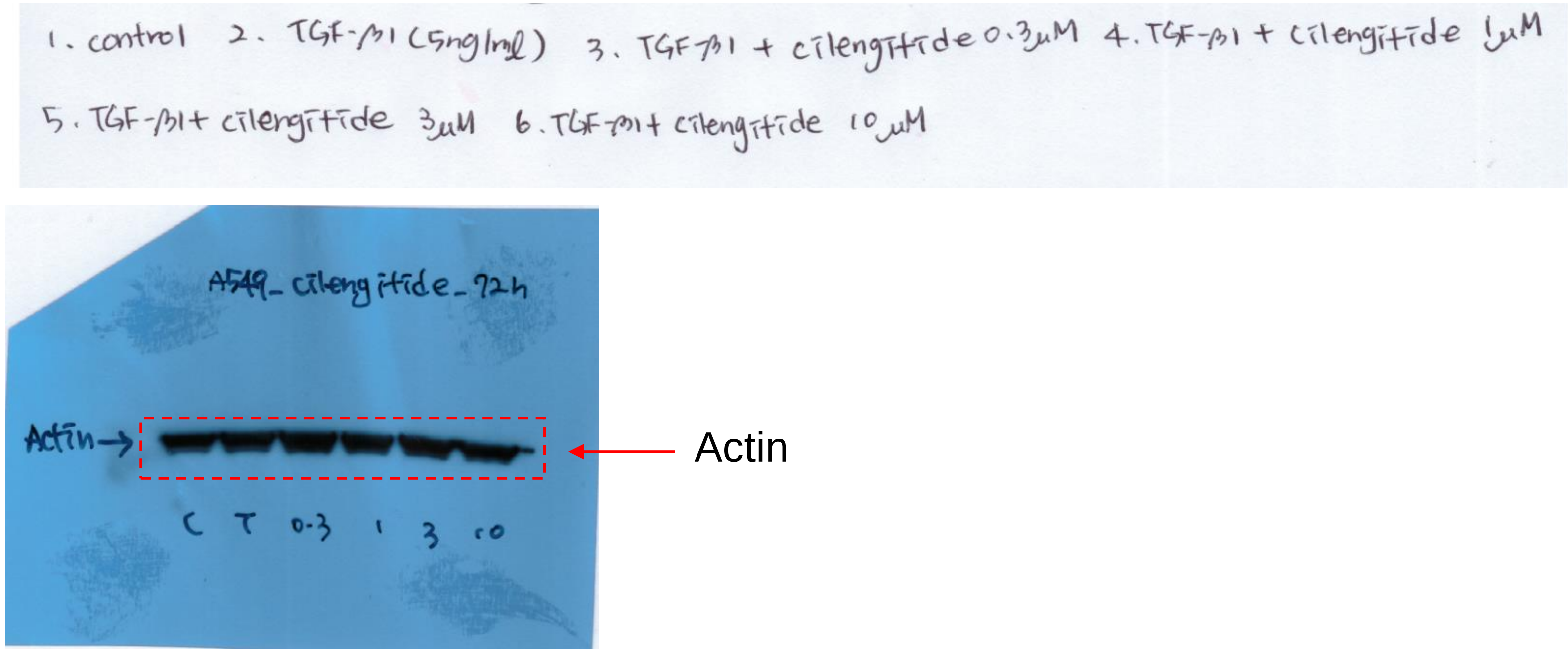

Fig. S7B

A549_Cilengitide_72 h

1. control 2. TGF-β1 (5ng/ml) 3. TGF-β1 + cilengitide 0.3μM 4. TGF-β1 + cilengitide 1μM
5. TGF-β1+ cilengitide 3μM 6. TGF-β1+ cilengitide 10μM

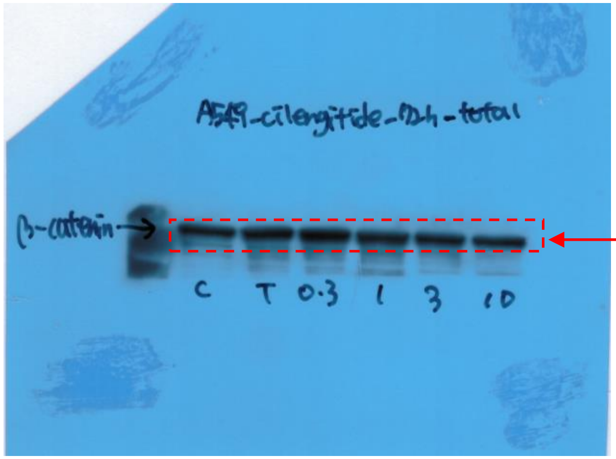

β-catenin

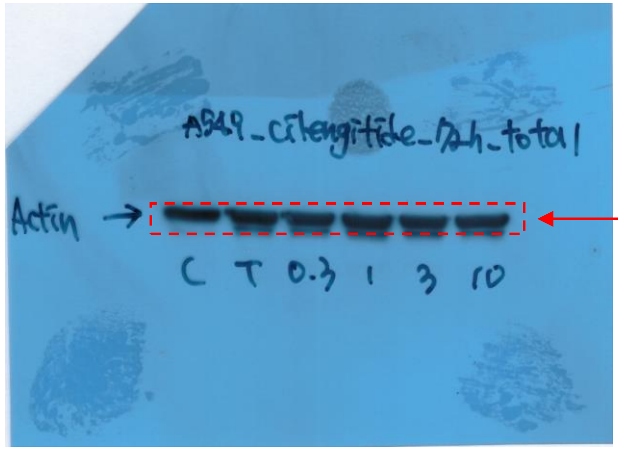

Actin

Fig. S8A

A549_Combi (Gefitinib + Cilengitide)

1. control 2. TGF-β1 (5ng/ml) 3. TGF-β1 + Gefitinib 1uM 4. TGF-β1 + cilengitide 3uM

5. TGF-β1 + Gefitinib 1uM + cilengitide 3uM

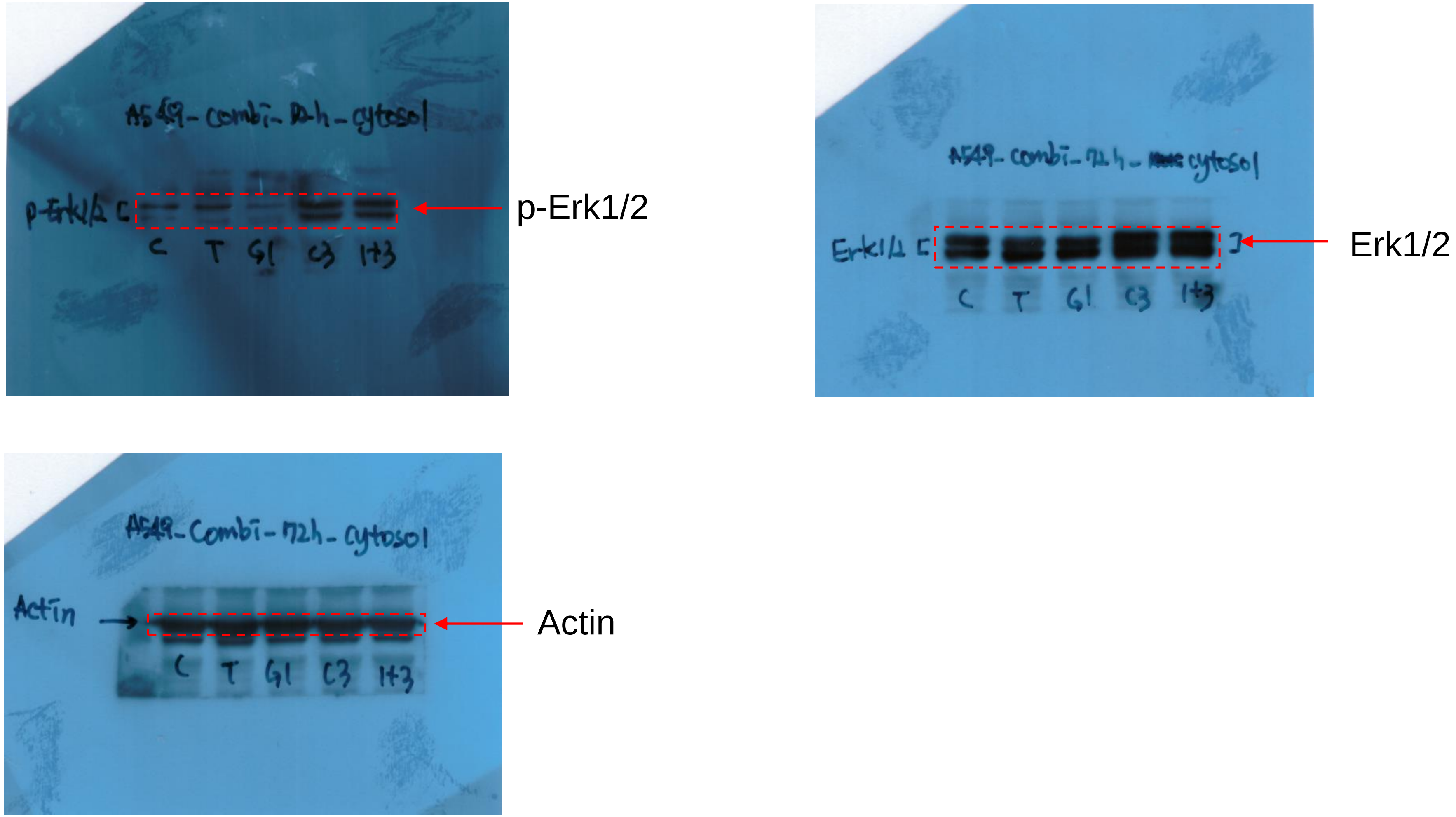

Fig. S8B

A549_Combi (Gefitinib + Cilengitide)

1. control 2. TGF-β1 (5ng/ml) 3. TGF-β1 + Gefitinib 1uM 4. TGF-β1 + cilengitide 3uM
5. TGF-β1 + Gefitinib 1uM + cilengitide 3uM

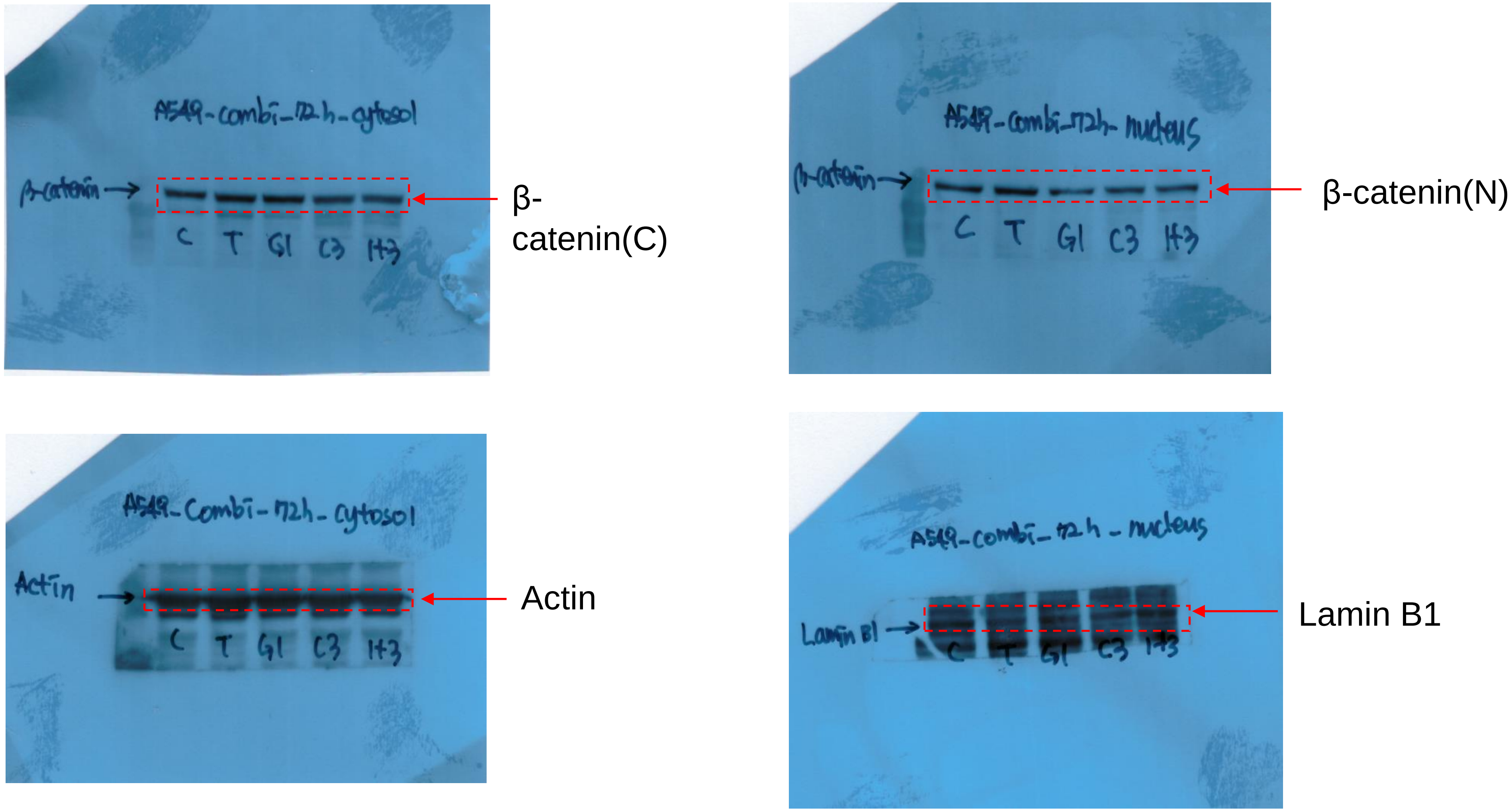

Supplement: Supplementary file 1 [file datasheet1.pdf]
