# Supplementary material for: Cyclic RGD Pentapeptide Cilengitide Enhances Efficacy of Gefitinib on TGF-β1-Induced Epithelial-to-Mesenchymal Transition and Invasion in Human Non-Small Cell Lung Cancer Cells
Source: Front Pharmacol. 2021 Mar 24;12:639095. doi: 10.3389/fphar.2021.639095 (PMC8104086; doi:10.3389/fphar.2021.639095)
Supplement: Supplementary file 2 [file datasheet2.docx]

**Figure S1.** Synthetic scheme for cilengitide, c(RGDfV) **4**. Protected linear pentapeptide **1** bound to the resin was synthesized by the Fmoc solid phase peptide synthesis (SPPS) method. Linear peptide **2** was cleaved from the resin without affecting other protecting groups by acetic acid/TFE/CH_2_Cl_2_ (1:1:3 ratio) solution. Finally, cyclic pentapeptide c(RGDfV) **4** was obtained by head-to tail cyclization under T3P, TEA, DAMP, and elimination of the protecting group by TFA:H_2_O=19:1(v/v).


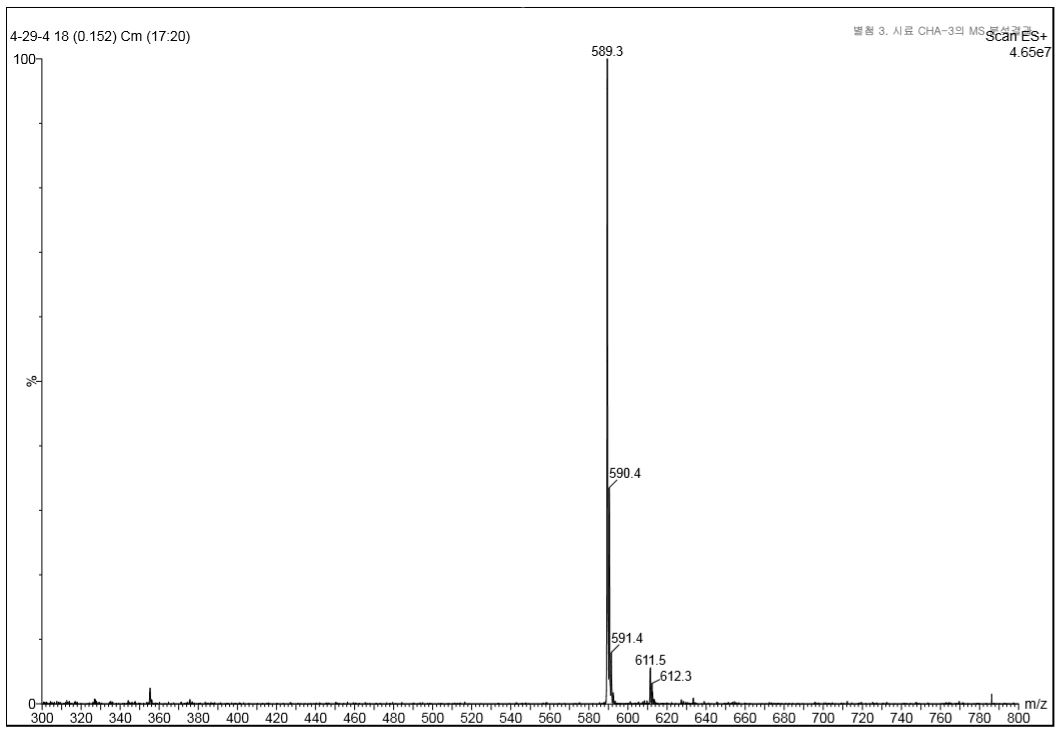


**Figure S2.** LC-mass spectrum of cilengitide. Mw calcd. 588.6, obsd. 589.3.


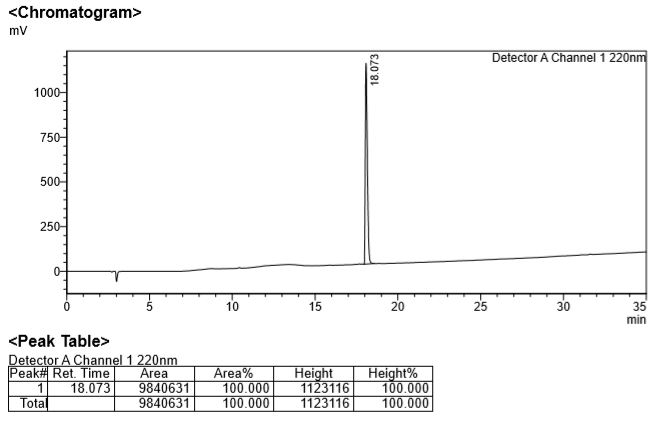


**Figure S3.** HPLC spectrum of cilengitide.

**Experimental Section**

**Mass spectrometry**

Cilengitide masses were determined on a Waters ACQUITY UPLC H-Class/SQD2.

**HPLC spectrometry**

HPLC analysis of cilengitide was conducted in H_2_O solution (1 mg/mL) using a Shimadzu HPLC 2030 model with a Vydac 218TP C18 column (5 μm, 4.6 × 250 mm). A buffer was 0.1% TFA in H_2_O and B buffer 0.1% TFA in CH_3_CN. The flow rate was 1 mL/min with a gradient of 0%-3% B in 3 min, 3%-60% B in 33 min, and 60% B in 35 min.


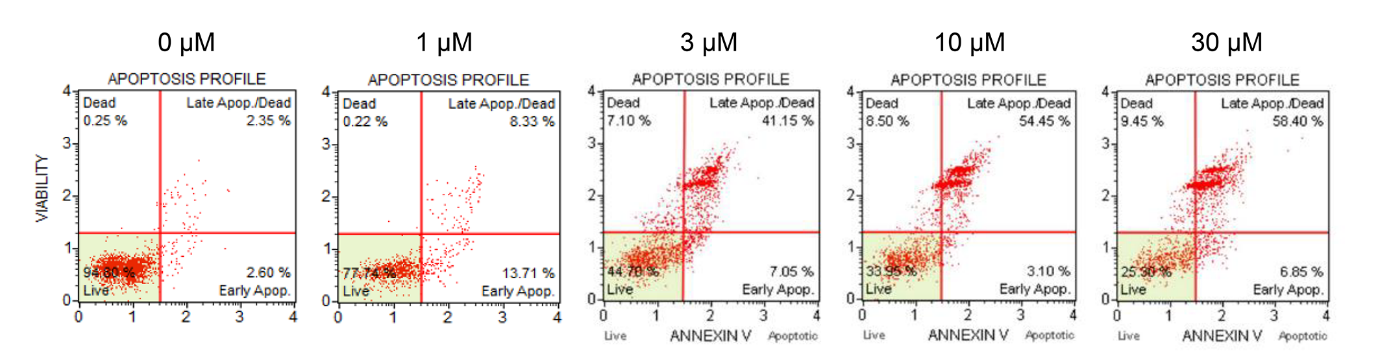


**Figure S4.** Cilengitide induces apoptosis. A549 cells were incubated with cilengitide for 24 h, and then the populations of apoptotic and dead cells were analyzed using the MUSE Cell Analyzer.


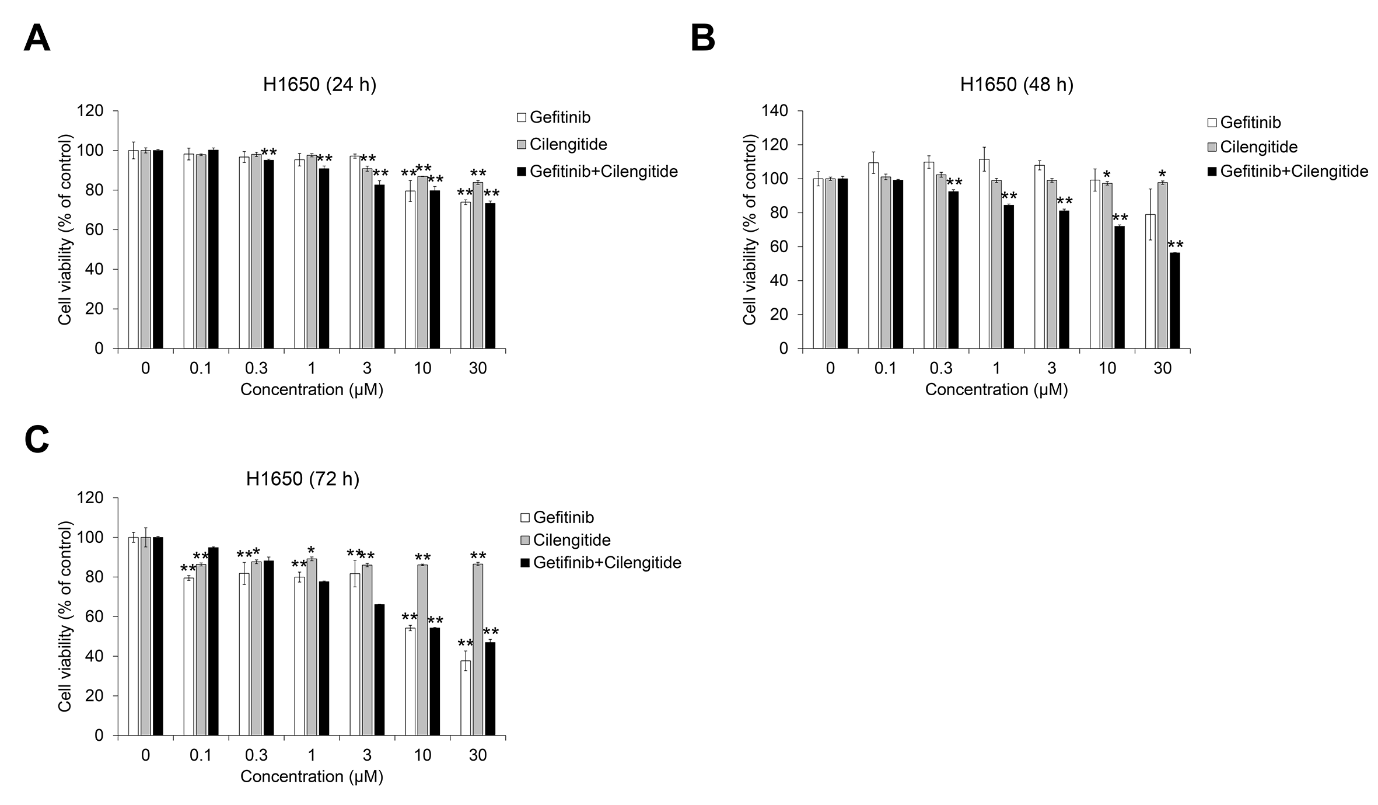


**Figure S5.** Cell viability of gefitinib, cilengitide, and combined treatment in NSCLC H11650 cells. H1650 cells were treated with gefitinib and/or cilengitide for 24–72 h. Cell viability was measured by the CCK-8 assay. Experiments were performed in triplicate. Data represent mean ± SD. * *p* < 0.05, ** *p* < 0.01 versus untreated control.


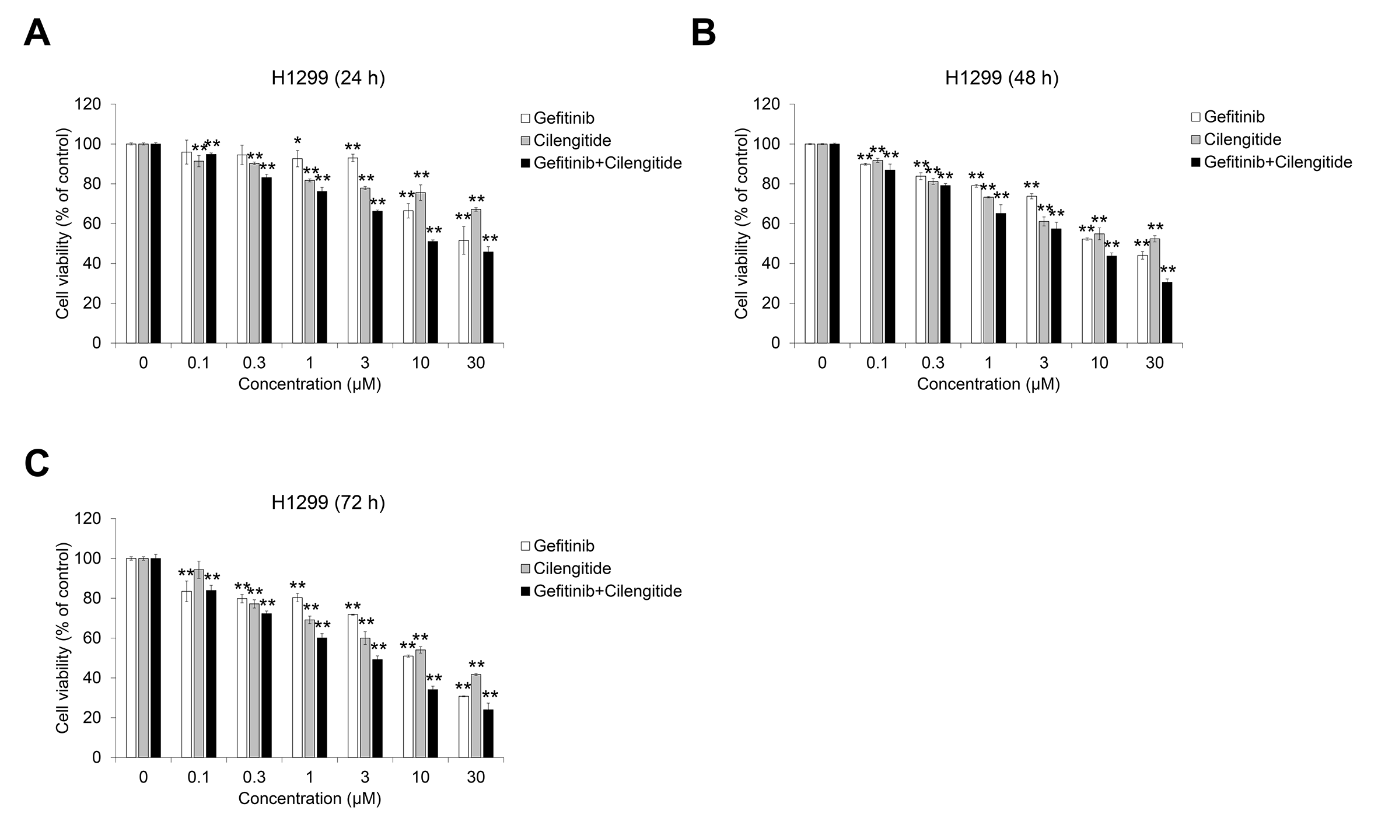


**Figure S6.** Cell viability of gefitinib, cilengitide, and combined treatment in NSCLC H1299 cells. H1299 cells were treated with gefitinib and/or cilengitide for 24–72 h. Cell viability was measured by the CCK-8 assay. Experiments were performed in triplicate. Data represent mean ± SD. * *p* < 0.05, ** *p* < 0.01 versus untreated control.


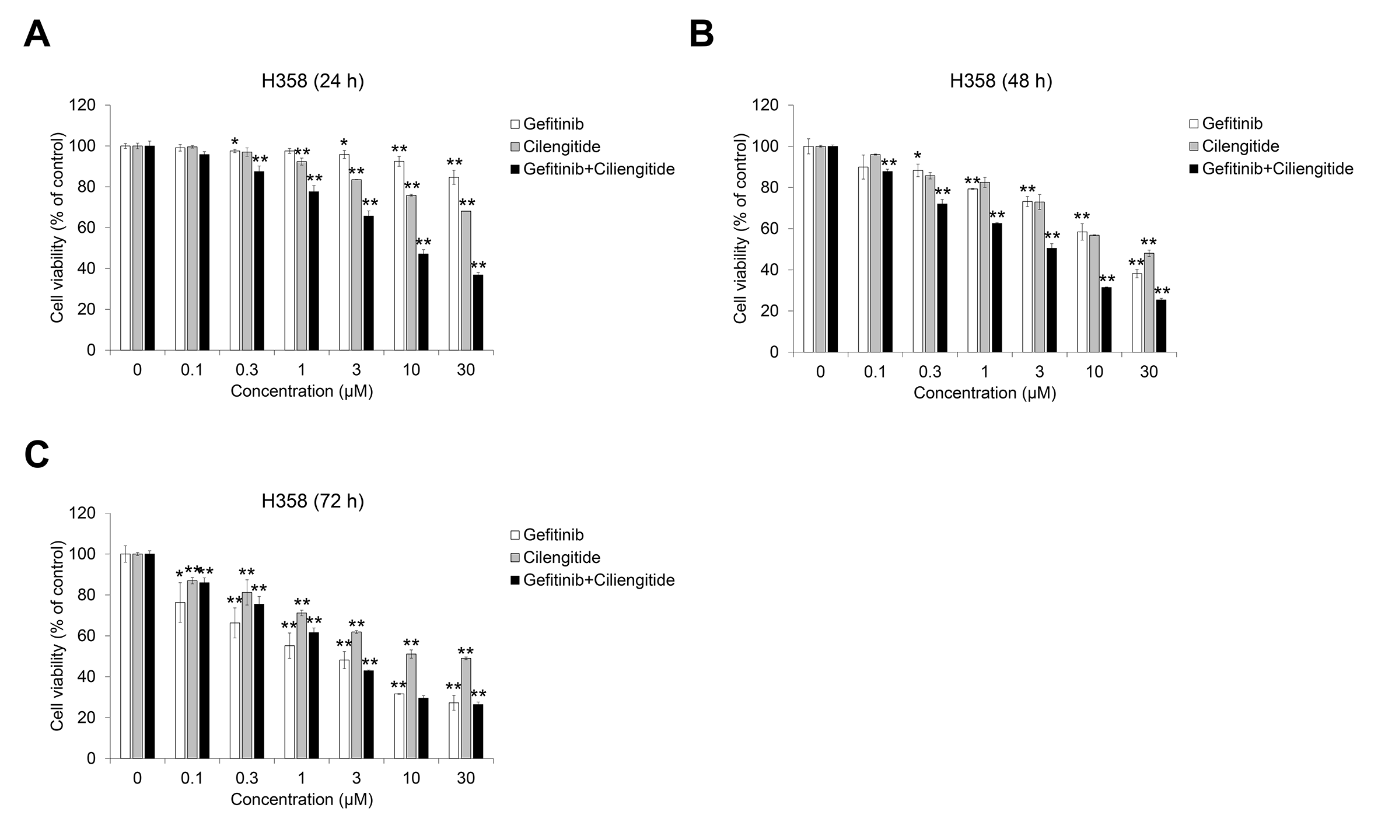


**Figure S7.** Cell viability of gefitinib, cilengitide, and combined treatment in NSCLC H358 cells. H358 cells were treated with gefitinib and/or cilengitide for 24–72 h. Cell viability was measured by the CCK-8 assay. Experiments were performed in triplicate. Data represent mean ± SD. * *p* < 0.05, ** *p* < 0.01 versus untreated control.


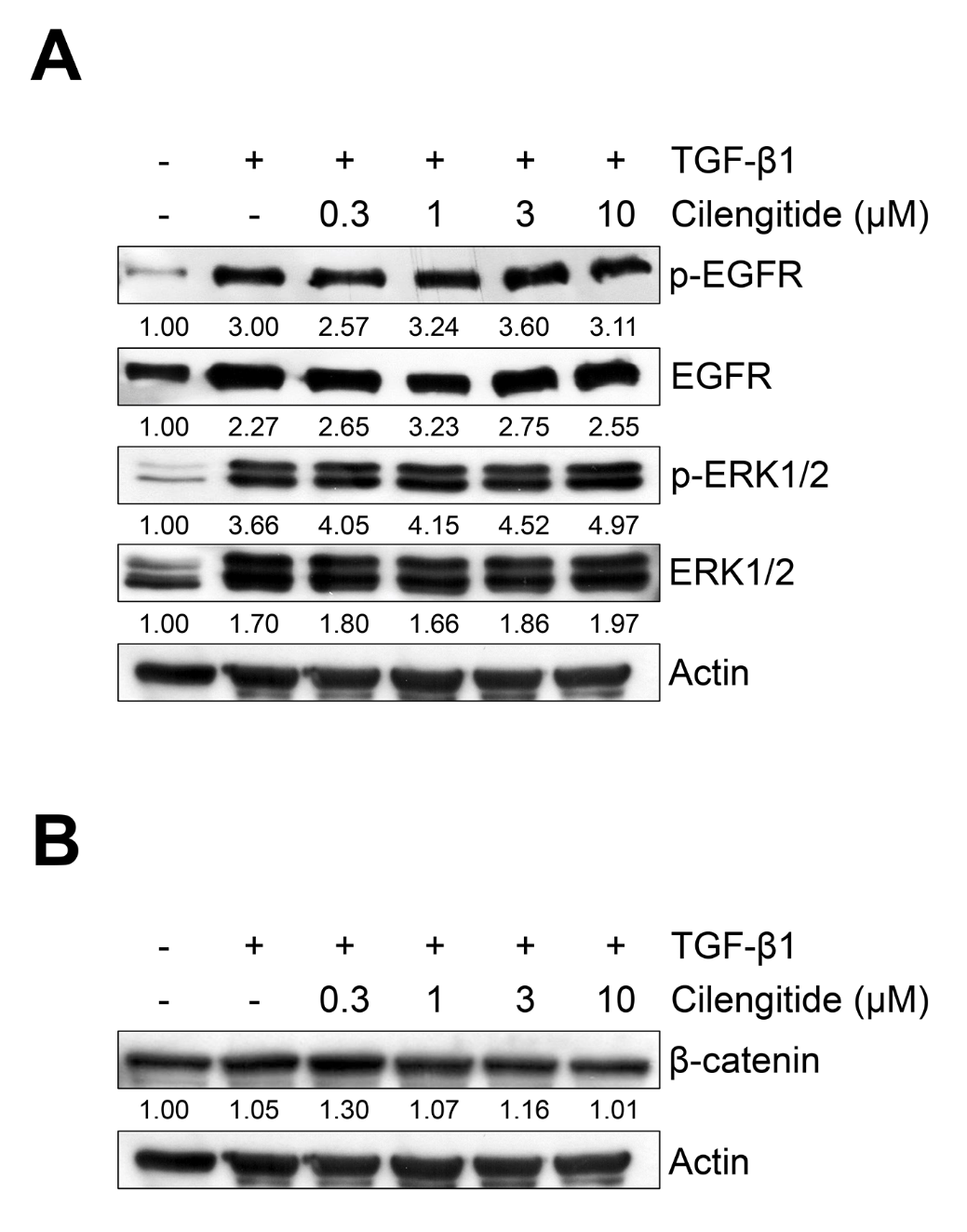


**Figure S8.** The effect of cilengitide on TGF-β1-induced non-Smad signaling pathway-related protein expression in A549 cells. Serum-deprived A549 cells were treated with TGF-β1 (5 ng/mL) and cilengitide for 72 h. Protein expression was measured by Western blot analysis. Actin was used as a loading control.


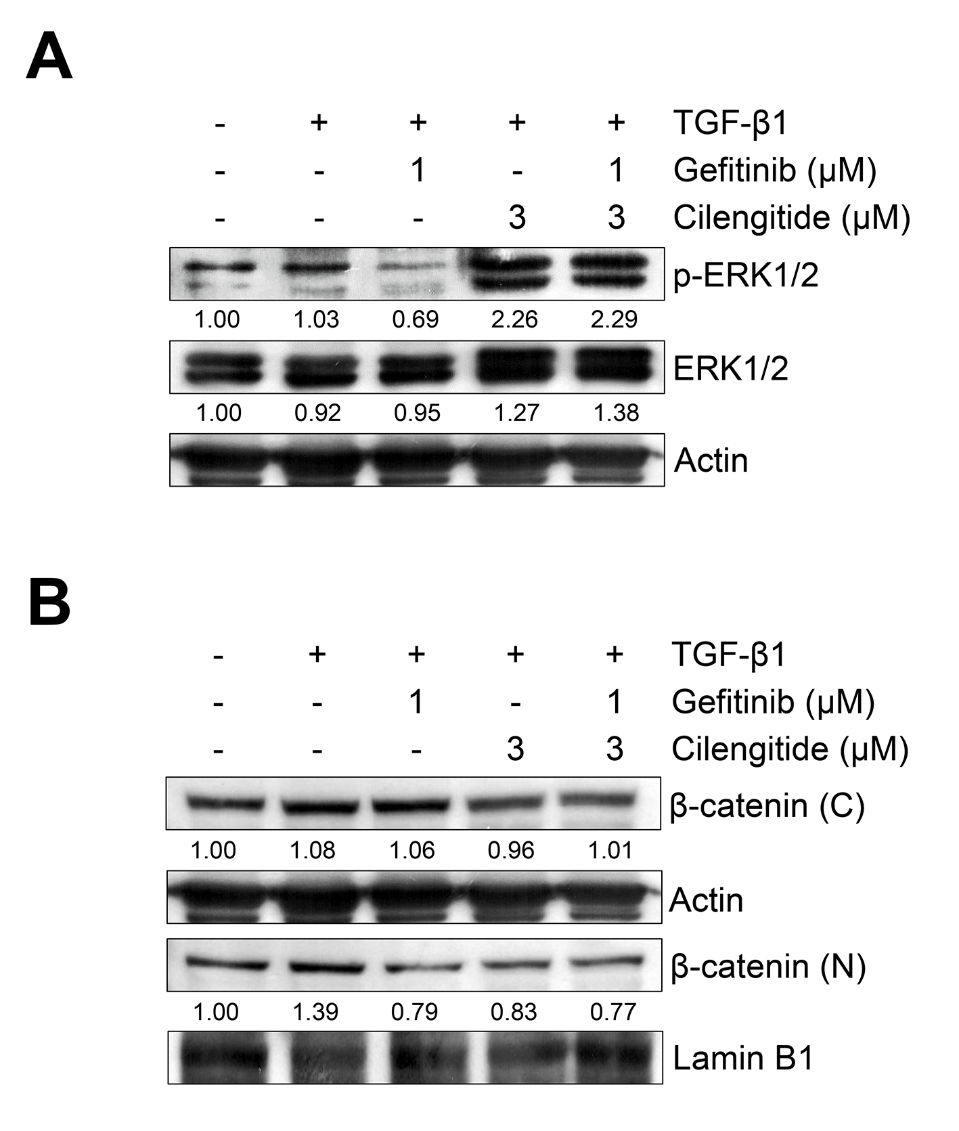


**Figure S9.** The effect of combined treatment with gefitinib and cilengitide on TGF-β1-induced non-Smad signaling pathway-related protein expression in A549 cells. Serum-deprived A549 cells were treated with TGF-β1 (5 ng/mL), gefitinib (1 μM), and cilengitide (3 μM) for 72 h. Cytosolic (C) or nuclear (N) protein expression was measured by Western blot analysis. Actin (C) and lamin B1 (N) were used as loading controls.

**Table S1.** Sequences of primers used in this study.

| **Target gene** | **Forward (5’ – 3’)** | **Reverse (5’ – 3’)** |
| --- | --- | --- |
| *TWIST1* | ATTCAGACCCTCAAGCTGGC | GAGAGGGGAGGAAATCGAG |
| *SNAI1* | ACCTCCAGACCCACTCAGAT | GCAGAGGACACAGAACCAGA |
| *CDH1* | TCCGAAGCTGCTAGTCTGAG | CTCAAGGGAAGGGAGCTGAA |
| *CDH2* | CCCACAGCTCCACCATATGA | TTCAGTCATCACCTCCACCA |
| *VIM* | CGCCAACTACATCGACAAGG | GGCTTTGTCGTTGGTTAGCT |
| *GAPDH* | GAGTCAACGGATTTGGTCGT | GATCTCGCTCCTGGAAGATG |

**Table S2.** Combination index (CI) values for the two-drug combination against A549 cell viability.

| Cell line | Incubation time (h) | Gefitinib (μM) | Cilengitide (μM) | CI value |
| --- | --- | --- | --- | --- |
| H1650 | 24 h | 0.1 | 0.1 | 1.0932 |
|  |  | 0.3 | 0.3 | 0.4454 |
|  |  | 1 | 1 | 0.4353 |
|  |  | 3 | 3 | 0.2136 |
|  |  | 10 | 10 | 0.4148 |
|  |  | 30 | 30 | 0.4227 |
|  | 48 h | 0.1 | 0.1 | 18.3808 |
|  |  | 0.3 | 0.3 | 1.8295 |
|  |  | 1 | 1 | 0.4263 |
|  |  | 3 | 3 | 0.5862 |
|  |  | 10 | 10 | 0.1999 |
|  |  | 30 | 30 | 0.0257 |
|  | 72 h | 0.1 | 0.1 | 0.2488 |
|  |  | 0.3 | 0.3 | 0.2254 |
|  |  | 1 | 1 | 0.1967 |
|  |  | 3 | 3 | 0.1841 |
|  |  | 10 | 10 | 0.2096 |
|  |  | 30 | 30 | 0.3276 |
| A549 | 24 h | 0.1 | 0.1 | 1.1024 |
|  |  | 0.3 | 0.3 | 10.0596 |
|  |  | 1 | 1 | 1.278E-09 |
|  |  | 3 | 3 | 9.27E-13 |
|  |  | 10 | 10 | 1.10E-19 |
|  |  | 30 | 30 | 1.55E-22 |
|  | 48 h | 0.1 | 0.1 | 0.4465 |
|  |  | 0.3 | 0.3 | 0.4095 |
|  |  | 1 | 1 | 0.4208 |
|  |  | 3 | 3 | 0.5971 |
|  |  | 10 | 10 | 0.7592 |
|  |  | 30 | 30 | 0.7081 |
|  | 72 h | 0.1 | 0.1 | 1.4553 |
|  |  | 0.3 | 0.3 | 0.8107 |
|  |  | 1 | 1 | 0.5699 |
|  |  | 3 | 3 | 0.6080 |
|  |  | 10 | 10 | 0.4652 |
|  |  | 30 | 30 | 0.4658 |
| H358 | 24 h | 0.1 | 0.1 | 0.9346 |
|  |  | 0.3 | 0.3 | 0.3180 |
|  |  | 1 | 1 | 0.2130 |
|  |  | 3 | 3 | 0.1646 |
|  |  | 10 | 10 | 0.1051 |
|  |  | 30 | 30 | 0.1306 |
|  | 48 h | 0.1 | 0.1 | 1.1451 |
|  |  | 0.3 | 0.3 | 0.3225 |
|  |  | 1 | 1 | 0.3677 |
|  |  | 3 | 3 | 0.3139 |
|  |  | 10 | 10 | 0.1322 |
|  |  | 30 | 30 | 0.1826 |
|  | 72 h | 0.1 | 0.1 | 1.1929 |
|  |  | 0.3 | 0.3 | 0.7758 |
|  |  | 1 | 1 | 0.5776 |
|  |  | 3 | 3 | 0.2973 |
|  |  | 10 | 10 | 0.2532 |
|  |  | 30 | 30 | 0.5287 |
